# RIG‐I Mediated Neuron‐Specific IFN Type 1 Signaling in FUS‐ALS Induces Neurodegeneration and Offers New Biomarker‐Driven Individualized Treatment Options for (FUS‐)ALS

**DOI:** 10.1002/advs.202417135

**Published:** 2026-01-28

**Authors:** Marcel Naumann, Theresa M. Wierschin, Stefanie Kretschmer, Banaja P. Dash, Aaron Held, Andrea Salzinger, Kevin Peikert, Anže Karlek, Hannes Glaß, Dajana Großmann, René Günther, Susanne Petri, Annekathrin Rödiger, David Brenner, Francisco Pan‐Montojo, Eleonora Aronica, Markus Kipp, Vitaly Zimyanin, Jared Sterneckert, Torsten Grehl, Noah D. Seebacher, Tobias M. Böckers, Alberto Catanese, Brian J. Wainger, Patrick Oeckl, Min Ae Lee‐Kirsch, Andreas Hermann

**Affiliations:** ^1^ Translational Neurodegeneration Section “Albrecht Kossel” Department of Neurology University Medical Center Rostock University of Rostock Rostock Germany; ^2^ Department of Pediatrics, Medizinische Fakultät Carl Gustav Carus Technische Universität Dresden Dresden Germany; ^3^ Department of Neurology Sean M. Healey & AMG Center for ALS Harvard Medical School Massachusetts General Hospital Boston Massachusetts USA; ^4^ Center For Transdisciplinary Neurosciences Rostock (CTNR) University Medical Center Rostock University of Rostock Rostock Germany; ^5^ Department of Neurology University Hospital Carl Gustav Carus At Technische Universität Dresden Dresden Germany; ^6^ German Center for Neurodegenerative DIseases (DZNE) Dresden Dresden Germany; ^7^ Department of Neurology Hannover Medical School Hannover Germany; ^8^ Department of Neurology Jena University Hospital Jena Germany; ^9^ Department of Neurology University Hospital Ulm Ulm Germany; ^10^ Dept. of Psychiatry and Psychotherapy at the Klinikum LMU, Munich Germany and Neurologische Klinik Sorpesee Sundern Germany; ^11^ Neurosciences Area Biogipuzkoa Health Research Institute Donostia/San Sebastian Spain; ^12^ CIBERNED ISCIII (CIBER, Carlos III Institute, Spanish Ministry of Sciences and Innovation) Madrid Spain; ^13^ Amsterdam UMC University of Amsterdam Department of (Neuro)Pathology Amsterdam Neuroscience Amsterdam The Netherlands; ^14^ Rostock University Medical Center Institute of Anatomy Rostock Germany; ^15^ Department of Molecular Physiology and Biological Physics School of Medicine University of Virginia Charlottesville Virginia USA; ^16^ Center For Membrane and Cell Physiology School of Medicine University of Virginia Charlottesville Virginia USA; ^17^ Center For Regenerative Therapies TU Dresden (CRTD) and the Medizinische Fakultät Carl Gustav Carus Technische Universität Dresden Dresden Germany; ^18^ Department of Neurology Alfried Krupp Hospital Essen Germany; ^19^ Institute of Anatomy and Cell Biology University of Ulm Ulm Germany; ^20^ German Center for Neurodegenerative Diseases (DZNE) Ulm Ulm Germany; ^21^ Institute of Neuroanatomy University Clinic Aachen Aachen Germany; ^22^ Department of Anesthesiology Critical Care and Pain Medicine Massachusetts General Hospital Boston Massachusetts USA; ^23^ Broad Institute of Harvard University and MIT Cambridge Massachusetts USA; ^24^ University Centre for Rare Diseases University Hospital Carl Gustav Carus Technische Universität Dresden Dresden Germany; ^25^ German Center for Child and Adolescent Health (DZKJ), partner site Leipzig/Dresden Dresden Germany; ^26^ German Center for Neurodegenerative Diseases (DZNE) Rostock/Greifswald Rostock Germany

**Keywords:** cGAS‐STING pathway, double‐stranded RNA, pathogen‐associated molecular patterns, RIG‐I, RIG‐I like receptors, RNA‐sequencing, type 1 interferon

## Abstract

Recent research demonstrated activation of the innate immune system in ALS models. This pathway can be activated by cGAS‐STING sensing of cytosolic DNA that accumulates as a result of chronic DNA damage and defective mitochondria, both of which was identified as pathology in FUS‐ALS. Therefore, we analyzed innate immune pathways in FUS‐ALS, which revealed upregulation of interferon‐stimulated genes (ISGs) and activation of the TBK1‐IRF3 pathway in FUS^mut^ iPSC‐derived spinal motor neurons (sMNs). Accumulation of cytosolic dsRNA and its sensor RIG‐I, but not MDA5, was found to be significantly upregulated in FUS^mut^ sMNs, which was abolished upon siRNA‐mediated knockdown of RIG‐I. RIG‐I was highly expressed in FUS‐ALS post‐mortem α‐MNs. IFN treatment of FUS^wt^ sMNs phenocopied the axonal degeneration of FUS^mut^ sMNs. Mitochondrial transcription, a known source of dsRNA, was found to be upregulated in compartmental axonal RNAseq analysis and its inhibition reduced ISGs in FUS‐ALS sMNs. The JAK‐STAT inhibitor ruxolitinib alleviated the upregulated ISG expression and reversed the axonal degeneration of sMNs. Finally, we analyzed ISG expression in peripheral blood from 18 FUS‐ALS patients, eight of whom had a significantly elevated interferon signature. RIG‐I‐mediated innate immune activation in sMNs may be an interesting novel individualized biomarker‐driven therapeutic target in (FUS‐) ALS.

**A one‐sentence summary of your paper**: RIG‐I‐mediated innate immune activation is found in FUS‐ALS spinal motor neurons caused by cytosolic dsRNA accumulation due to mitochondrial transcriptional activation and is amenable to JAK‐STAT inhibition and might thus be an interesting novel individualized biomarker‐driven therapeutic approach in (FUS‐) ALS.

## Graphical abstract

Using iPSC‐derived motoneurons and postmortem tissue from FUS‐ALS patients, it is demonstrated that increased mitochondrial transcription leads to elevated cytosolic double‐stranded RNA (dsRNA) levels. This aberrant accumulation activates a RIG‐I–dependent innate immune response leading to neurodegeneration, which is amenable for FDA‐ and EMA‐approved JAK inhibitors providing an interesting novel individualized biomarker‐driven therapeutic approach for (FUS‐) ALS.

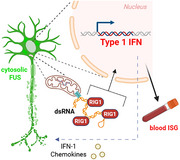

## 1 Introduction

Despite decades of intensive research, our understanding of the specific pathomechanisms of selective motor neuron degeneration in amyotrophic lateral sclerosis (ALS), a devastating neurodegenerative disease, is incomplete. In addition to perturbations in proteostasis, autophagy, and endoplasmic reticulum (ER) stress, among others, (for review see [1]), there is growing evidence that innate immune pathways promote sterile neuroinflammation, which may contribute significantly to the pathomechanisms of ALS and other neurodegenerative diseases [2, 3]. In detail, mitochondrial distress and increased production of reactive oxygen species fatally synergize to induce nuclear or mitochondrial DNA damage and increase the permeability of the mitochondrial transition pores [4]. This leads to the translocation of damaged nuclear and/or mitochondrial DNA into the cytosol, which is recognized as “foreign” by conserved cellular mechanisms whose actual purpose is to elicit a response to get rid of pathogens after sensing their foreign DNA during infection. In neurodegenerative diseases, however, these mechanisms become suicidal. First, recognition of cytosolic double‐stranded DNA is mediated by cyclic guanosine monophosphate–adenosine monophosphate (cGAMP) synthase (cGAS), which activates the adaptor protein stimulator of interferon genes (STING) [5]. Second, double‐stranded RNA derived from damaged mitochondria is sensed by members of the Retinoic acid‐inducible gene I (RIG‐I)‐like receptor (RLR) family, such as RIG‐I (also known as DDX58), which interacts with the mitochondrial antiviral signaling protein (MAVS) [6]. Both adaptor proteins, STING and MAVS, recruit TANK‐binding kinase 1 (TBK1) and are phosphorylated by it at a conserved c‐terminal consensus motif [7], which is independent of other cellular functions of TBK1, such as in autophagy. In particular, this allows the recruitment and phosphorylation of interferon regulatory factor 3 (IRF3) [7, 8]. Phosphorylated IRF3, in turn, dimerizes and shuttles to the nucleus to induce the expression of interferon‐stimulated genes (ISGs), resulting in the activation of type I interferon (IFN‐1), stimulation of the TNF‐alpha pathway and the upregulation of several other inflammatory mediators, pro‐apoptotic genes and chemokines [7]. In unaffected tissues, there is a low or ‘tonic’ production of IFN‐1, which is essential for normal cellular function and host defense against microbial pathogens. This physiological homeostasis can be thrown out of balance by TBK1‐IRF3 signaling in, for example, senescent microglia, putting them in a reactive state that causes non‐cell autonomous neuronal neurotoxicity [2].

Importantly, C9orf72 mutations have been shown to cause over‐activation of the innate immune system in mouse models, cell culture systems and patient blood samples [9, 10, 11]. This has been experimentally attributed to activation of the TBK1‐IRF3 pathway. However, different primary mechanisms for promoting this pathway have been described: On the one hand, it was shown that loss of C9orf72 led to reduced clearance of STING by autophagy, resulting in chronic TBK1 stimulation [11]. On the other hand, bidirectional transcription of C9orf72 repeats was shown to generate sense and antisense mRNA with subsequent dsRNA formation. This was shown to trigger MDA5 activation leading to neuronal loss in C9orf72 HRE mutant mouse model systems and human iPSC‐derived sMNs [10]. In TARDBP mutant model systems, STING activation was identified as a result of mitochondrial DNA release due to TDP‐43‐mediated opening of the mitochondrial permeability transition pore (mPTP) [4]. In a SOD1 mutant ALS mouse model, mitochondrial misfolded SOD1 was found to promote mtDNA and RNA:DNA release into the cytosol independently of mPTP, resulting in STING activation [12]. Finally, recent evidence from human post‐mortem studies suggests an elemental, cell‐autonomous abundance of STING in cortical and spinal motoneurons in sporadic and some familial forms of ALS [13].

Overall, overactivity of the cGAS‐STING/RIG‐I and downstream TBK1‐IRF3 signaling pathways has recently been identified as a major contributor to neurodegeneration in genetic models of ALS. However, it remains unclear whether similar mechanisms are involved in FUS‐ALS. Previously, we and others have shown that FUS‐ALS spinal motor neurons (sMNs) accumulate DNA damage and depolarized axonal mitochondria [14, 15, 16]. Therefore, the IFN‐1 response may also be central to the pathogenesis of FUS ALS. There is considerable evidence that DNA damage may precede (axonal) neurodegeneration in several ALS models, including FUS [14, 17]. However, the relationship between the two remains to be elucidated. Some reports suggest that TNFα treatment induces axonal dysfunction [18] and imply a neurotoxic effect of chronic IFN1 exposure on neurons [19]. Therefore, we aimed to investigate the role of IFN1 signaling in FUS‐ALS to determine if there is a neuronal, cell‐autonomous activation of IFN1 signaling and, if so, through which specific pathway it is mediated and whether this IFN1 signaling might be a link between DNA damage and the observed axon degeneration. The canonical IFN1 pathway activates the Janus kinase (JAK) signal transducer and activator of transcription (STAT) pathway. Since JAK‐STAT inhibitors are already FDA‐approved for several non‐neurological diseases, we wanted to further investigate whether they could alleviate neurodegeneration in ALS and thus represent an interesting repurposing strategy for a biomarker‐driven FUS‐ALS therapeutic approach.

## 2 Results

### 2.1 Activation of Type I Interferon Response in iPSC‐derived FUS^mut^ Mutant Motor Neurons

**FIGURE 1:**
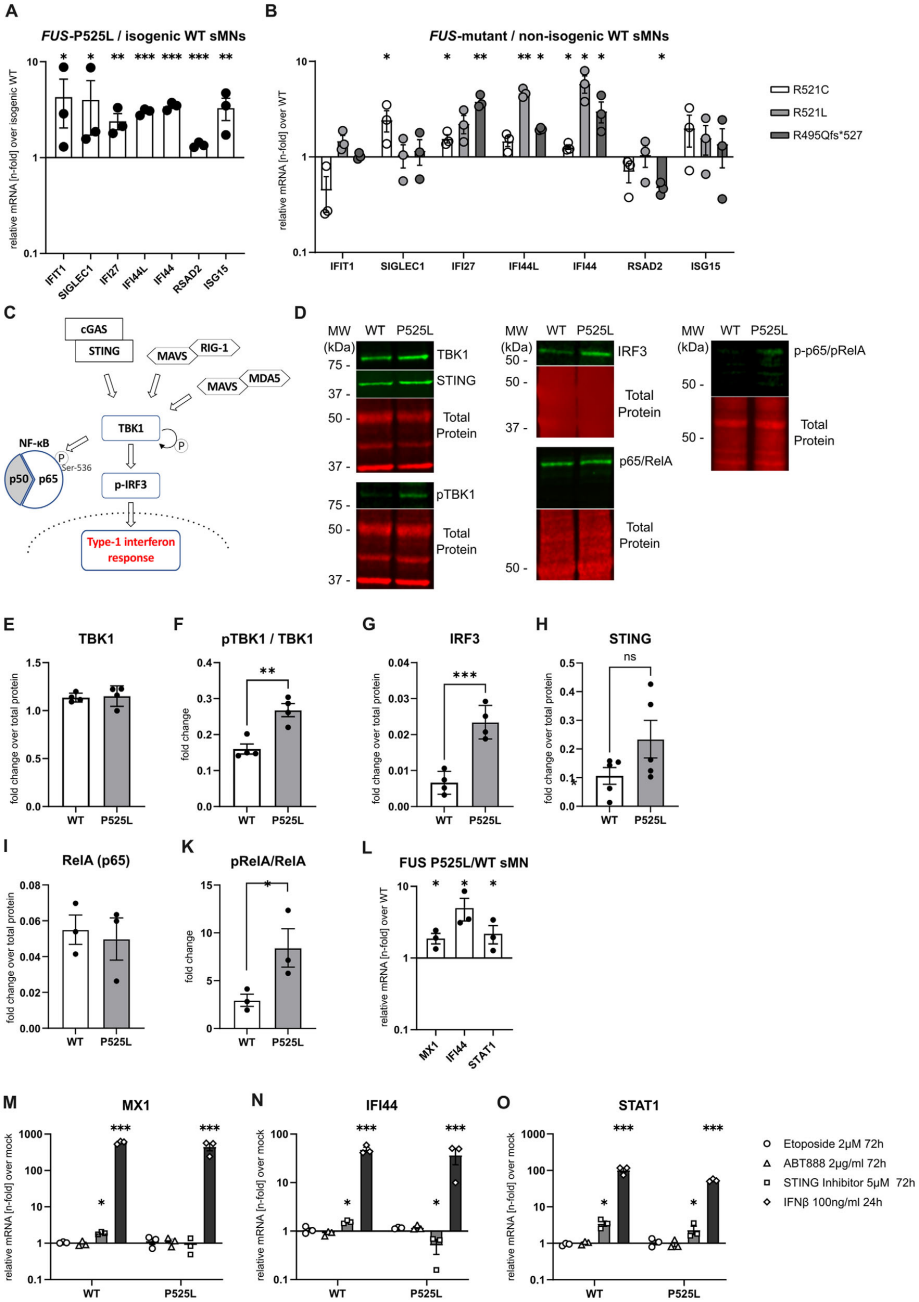
Innate immune activation in iPSC‐derived spinal motoneurons with mutations in *FUS*. (A,B) RT‐qPCR for a set of ISGs, as indicated on the x‐axis in sMNs with a FUS‐P525L mutation, normalized to an isogenic control line (A) and three other sMNs lines with distinct C‐terminal *FUS* mutations normalized to two non‐isogenic FUS^wt^ sMNs lines (B). Mann‐Whitney test for IFIT1 and SIGLEC1 expression in (A), for all other ISGs an unpaired t‐test was performed, respectively, n = 3 biological replicates. (C) sketch of the TBK1‐IRF3 pathway. TBK1 activation by either RIG‐I or cGAS sensing results in downstream transcriptional activation of a type‐1 IFN response following the translocation of phosphorylated IRF3 into the nucleus or phosphorylation of p65 at Ser‐536. (D) Cropped example western blot images including molecular weight markers indicating protein expression of markers of the TBK1‐IRF3 pathway in WT and isogenic FUS‐P525L sMNs. Full membranes are shown in Suppl. Figure S7 and cropped images are indicated by yellow rectangles. (E–H) Quantification of (D) normalized to a total protein staining, n = 4, unpaired t‐test. (I,K) Quantification of RelA and p‐RelA WB band intensity, n = 3, unpaired t‐test. (L) RT‐qPCR for selectively IFN‐1 dependent ISGs (MX1, IFI44, and STAT1) in FUS‐P525L sMNs normalized to isogenic control and (M‐O) influence of different treatment strategies on their expression normalized to mock treatment, one‐way ANOVA, Tukey's post hoc, n = 3 biological replicates. DNA‐damaging approaches (etoposide, PARP inhibitor ABT888) did not change their expression in FUS P525L or FUS WT sMN Similarly, the STING inhibitor H151 did not robustly lower the ISG expression in mutant sMNs. Treatment with IFN‐beta as a positive control.

Previously, we have shown that FUS‐ALS sMNs accumulate DNA damage and depolarized axonal mitochondria [14, 15]. This led us to hypothesize that there may be at least one mechanism that triggers an innate immune response, either through mitochondrial or nuclear DNA leakage. To address whether FUS mutations lead to innate immune activation within the neurons themselves, we assessed IFN1 pathway stimulation by measuring a set of interferon‐stimulated genes (ISGs) (Figure 1A,B) by RT‐qPCR under baseline conditions. This was performed in iPSC‐derived sMNs cultures devoid of glial cells [14], comparing the FUS‐P525L mutation with an isogenic control (Figure 1A). These cell lines were previously edited by CRISPR‐Cas9n to create an isogenic condition and add GFP to the C terminus of FUS in both cell lines [14]. Three additional (patient‐derived) FUS mutant iPSC‐derived sMNs cell lines (R521C, R521L, R495Qfs*527) were analyzed for ISG expression, which was normalised to two non‐isogenic FUS^WT^ control sMNs lines (Figure 1B). All lines were previously established and published, including negative genetic testing for C9ORF72, FUS, TARDBP, and SOD1 for the WT cell lines [14]. Furthermore, RNA‐Seq and microarray data did not show evidence for the expression of glial markers in the different cell lines (Suppl. Figure S1). In contrast, robust expression of neuronal markers was observed in all cell lines.

From our previous study, we knew that significant mitochondrial phenotypes and DNA damage accumulation were not present until at least 3 weeks during neuronal maturation, which is why the ISG measurement was performed at this time point. For FUS‐P525L sMNs, there was a robust upregulation for all ISGs examined (Figure 1A). For the non‐isogenic lines, there was still a significant upregulation of some ISGs (Figure 1B). Additionally, we used an independent isogenic pair, namely the KOLF cell line with a heterozygous FUS‐P525L mutation and compared it to its isogenic WT. Unlike the previously used cells, these were differentiated into motoneurons by a doxycycline‐inducible NGN2‐ISL1‐LHX3 (hNIL) piggyBac overexpression construct [20]. As demonstrated previously with different cell lines, this approach enables a high differentiation efficiency, with more than 95% of all cells expressing Hb9 and ISL1/2 (Suppl. Figure S2A,B). Of note, there was no evidence for the presence of GFAP expressing cells (Suppl. Figure S2B,D). Instead, we found robust expression of the early sMN markers MNX1 and ISL1 by qPCR. Despite the different cell resource and differentiation protocols, KOLF P525L‐het hNIL‐sMN also showed a significant upregulation of ISGs compared to WT isogenic control hNIL‐sMN (Suppl. Figure S2E).

We then tested for activation of the TBK1‐IRF3 pathway, based on the hypothesis that this pathway might be responsible for the observed ISG stimulation. As shown in the sketch in Figure 1C, the TBK1‐IRF3 pathway acts as a common trunk of several upstream signaling pathways that function in sensing different pathogen‐associated molecular patterns (PAMPs). We further focused on the isogenic P525L and corresponding WT sMNs lines as they showed the most homogeneous IFN1 pathway activation at the mRNA level. As expected, there was no difference in the baseline level of TBK1 in both FUS^mut^ and FUS^WT^ sMNs (Figure 1E). However, we found a significant increase in Ser172 TBK1 phosphorylation (i.e. activation of TBK1) in mutant sMNs (Figure 1F). In our hands, neither the p‐IRF Western blot nor the immunofluorescence staining of nuclear enrichment of p‐IRF3 with suitable positive controls worked properly. However, mutant sMNs inherently expressed higher levels of IRF3 (Figure 1G), suggesting pathway activation, but we saw no significant difference in the protein level of STING (Figure 1H). These phenotypes were also reproducible in the KOLF‐P525L‐het hNIL‐sMNs (Suppl. Figure S3A–C) and in the FUS‐R495Qfs*527 sMNs where we found increased pTBK1/TBK1 rations compared to WT (Suppl. Figure S4C,D).

In addition, we found significantly increased p65 phosphorylation, consistent with NFκB activation (Figure 1I–K).

### 2.2 IFN‐1 Response in iPSC‐Derived FUS Mutant Motor Neurons Is Independent of DNA Damage and STING Signaling

We and others have shown that the role of FUS in the DNA damage response is downstream and dependent on poly (ADP‐)ribose polymerase 1 activation [14, 21]. To investigate whether DNA damage induction is a major source of ISG up‐regulation in our model, we treated cells for 3 days with 2 µM etoposide—a topoisomerase inhibitor known to induce DNA damage—or 2 µg/mL of the PARP inhibitor ABT888. For this experiment, we assessed the expression of ISGs (MX1, STAT1 and IFI44), which were significantly upregulated in FUS‐P525 sMNs (Figure 1L). Unexpectedly, DNA‐damaging treatments did not lead to an upregulation of these ISGs in either cell line (Figure 1M–O) although we knew from previous studies that this experimental design led to a robust DNA damage induction in identical experimental settings [14], as indicated by an increased number of γH2A.x nuclear foci following etoposide treatment (Suppl. Figure S5A). This suggests that activation of nuclear DNA damage may not be the source of the observed ISG activation.

Having found evidence for upregulation of the TBK1 pathway and ISG mRNA, we next sought to investigate the role of STING We found no significant difference in the baseline STING levels (Figure 1H). Next, we asked whether H151, an established small molecule inhibitor of STING blocking STING palmitoylation [22], could reduce the increased ISG activation found in FUS‐P525L sMNs, as previously shown in iPSC‐derived neurons with different genetic ALS mutations [13]. However, we did not observe a systematic decrease in ISG levels in the FUS^mut^ sMNs upon exposure to H151, some ISGs were even paradoxically upregulated after treatment Interferon‐beta was used to control for the extent of possible ISG stimulation in this cell type (Figure 1M–O).

### 2.3 Cytosolic dsRNA Is Increased in iPSC‐Derived FUS^mut^ Motoneurons

**FIGURE 2:**
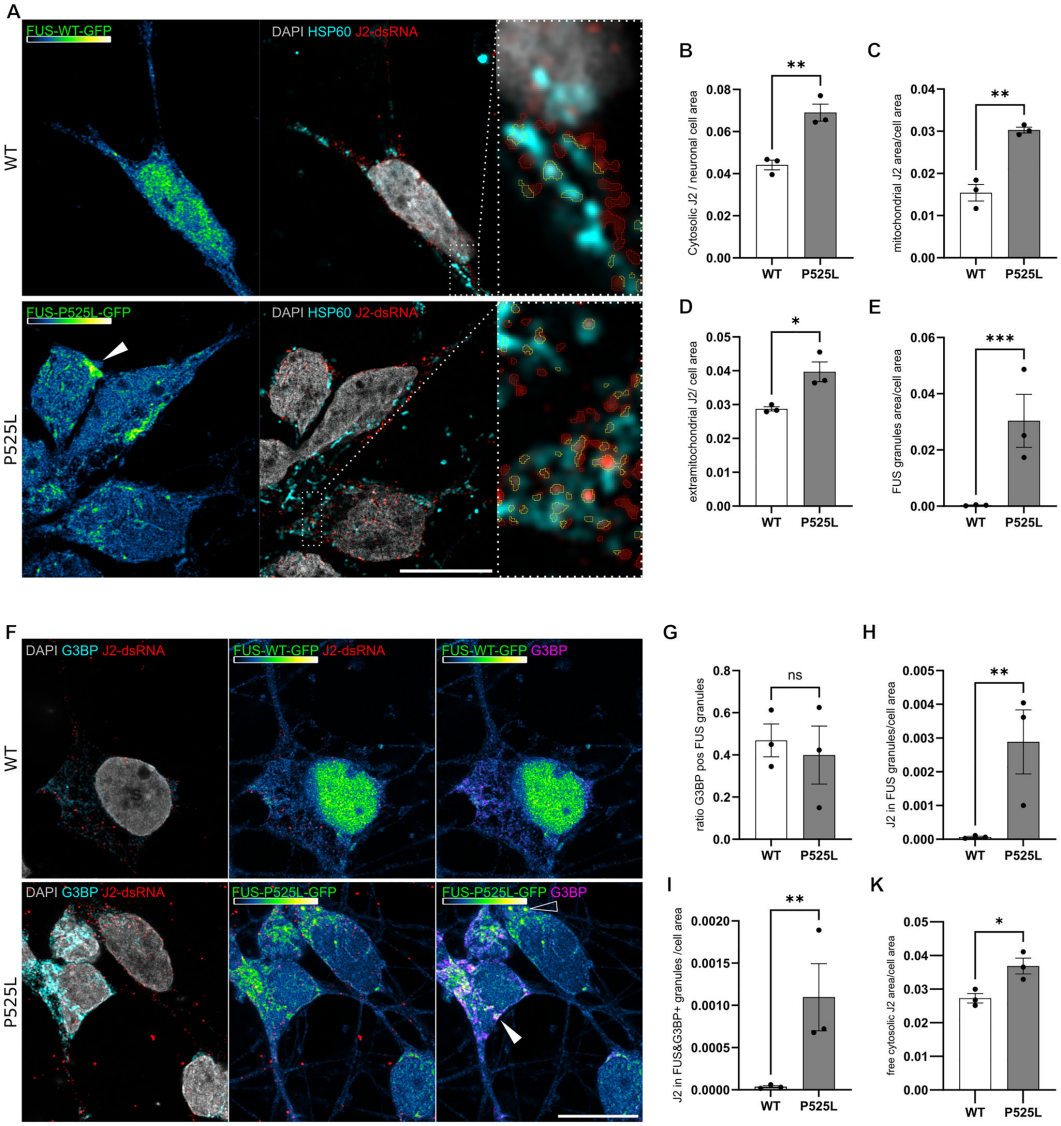
Assessment and quantification of cytosolic dsRNA in relation to mitochondrial compartments in iPSC‐derived sMNs. (A) IF image panel of WT and FUS‐P525L sMNs, scale bar 10 µM. Cells were stained against the mitochondrial matrix protein HSP60 and an anti‐dsRNA (J2) antibody (A). Nuclear staining was performed with DAPI. C‐terminally tagged FUS‐GFP indicates FUS presence in the different conditions and is visualized via the Green‐Fire‐Blue LUT in Fiji which allows to visualize the low intensity GFP cellular background. Note the nuclear loss of FUS‐P525L‐GFP. Dotted magnification boxes show the individual recognition of dsRNA (A) as region of interest (ROI) areas (yellow line) in Fiji, inside or outside of the HSP60 area. (B–D,K) Quantification of the cytosolic J2‐dsRNA ROI area was either done: within the single neuronal cell area (B) or within the single cell HSP60 compartment (C) and outside of it (D, extramitochondrial cytosolic) and outside of HSP60 and FUS granule area (K, free cytosolic). Normalization was as per whole cell area of the cell with the GFP background signal as a mask, respectively. Additionally, beta‐III‐tubulin was used when possible to create neuronal single‐cell masks (see Suppl. Figure S5C,6). (B–D) Significantly more dsRNA signal per cell was detected in FUS‐P525L sMNs compared to control in all conditions (t‐test, n = 3). (F) IF image panel of WT and FUS‐P525L sMNs indicating J2‐dsRNA in relation to G3BP as a marker of stress granules, scale bar 10 µM. Note the spontaneous formation of complex FUS‐GFP granules in FUS‐P525L sMNs (see E for quantification, Mann‐Whitney test, n = 3), which were partially double‐labeled with G3BP‐positive granules (filled white arrowhead), but not always (arrowhead). Around 40% of all FUS granules were G3BP double‐positive without difference between the cell lines (G). (H, I) Enrichment of J2 in FUS‐P525L‐GFP granules (H) and in double‐positive granules (I). (G‐K) Mann‐Whitney test, n = 3.

Alternative pathways for ISG activation include RIG‐I/MDA5 sensing of dsRNA species. We therefore hypothesized that there might be an overabundance of cytosolic dsRNA triggering the observed innate immune stimulation in FUS^mut^ sMNs. To address this question, super‐resolution AiryScan2 LSM microscopy was performed to identify the cytosolic dsRNA immunofluorescence signal using the well‐established J2 antibody, which detects dsRNA species of at least 40 bp. To separate free cytosolic dsRNA from mitochondrial dsRNA, we generated masks from the immunofluorescence signal of the mitochondrial marker HSP60. We quantified both the mitochondrial and non‐mitochondrial/free‐cytosolic compartments of the dsRNA immunofluorescence signal. This was performed in the FUS‐P525L‐GFP (Figure 2A) and KOLF P525L‐het hNIL‐sMNs (Suppl. Figure S6A–E) and compared to the respective isogenic control sMNs. Additionally, we repeated the experiment for the FUS‐R495Qfs*527 sMNs, which had a modest upregulation of ISGs (Figure 1B) and compared it to a non‐isogenic control sMNs (Suppl. Figure S6F–K). To demonstrate the quantification technique, the insets outlined by the dotted line in Figure 2A show the masks generated in Fiji that recognized the cytosolic dsRNA signal and were used to measure pixel area. For proper recognition of the cellular shape, single‐cell neuronal masks were created by using a beta‐III‐tubulin (Tuj) staining or – if not otherwise possible – the GFP background signal with neuronal morphology (Suppl. Figure S5C; Suppl. Figure S6A,F).

Mitochondria are known to be a source of dsRNA production in the cell, as sense and antisense transcripts of the circular genome can align to form dsRNA, which can trigger an MDA5‐dependent lethal IFN1 response when released into the cytosol [23]. We therefore measured dsRNA abundance using J2 immunofluorescence staining. We found a clear J2 signal inside and outside the mitochondria in FUS^wt^ and FUS^mut^ sMNs (Figure 2B–D, Suppl. Figure S5B, Suppl. Figure S6A–K) irrespective of the individual mutation or differentiation method. At baseline, we observed a significantly higher amount of mitochondrial J2 in FUS‐P525L mutant sMNs. Notably, we also identified a higher proportion of cytosolic J2‐dsRNA outside the mitochondria in FUS^mut^ sMNs (Figure 2C,D, Suppl. Figure S6B,C and Suppl. Figure S6H,I). Another important finding was the translocation of J2 to FUS granules. Consistent with previous reports, FUS‐ALS sMNs spontaneously produced FUS‐GFP granules (Figure 2A,E). Double immunostaining revealed that ∼45% of FUS‐GFP granules were double labelled with the stress granule marker G3BP1 (Figure 2F,G). While FUS P525L sMNs showed significantly more FUS‐GFP+ granules (Figure 2E), there was no difference in the proportion of G3BP1+/FUS‐gfp+ granules (Figure 2G). Notably, FUS P525L sMNs contained more dsRNA‐containing granules, both within G3BP1+/FUS‐gfp+ granules and within G3BP1‐/FUS‐gfp+ granules (Figure 2H,I). Subtraction of this granule J2 signal from extramitochondrial J2 yielded the free cytosolic J2 fraction (Figure 2K), which was significantly higher in FUS^mut^ sMNs. In conclusion, we show that FUS^mut^ sMNs have significantly increased dsRNA in mitochondria and stress granules (SGs), but it is also found in higher amounts freely and uncompartmentalized in the cytosol of untreated FUS^mut^ sMNs.

### 2.4 ISG Activation Is Mediated by RIG‐I Signaling

**FIGURE 3:**
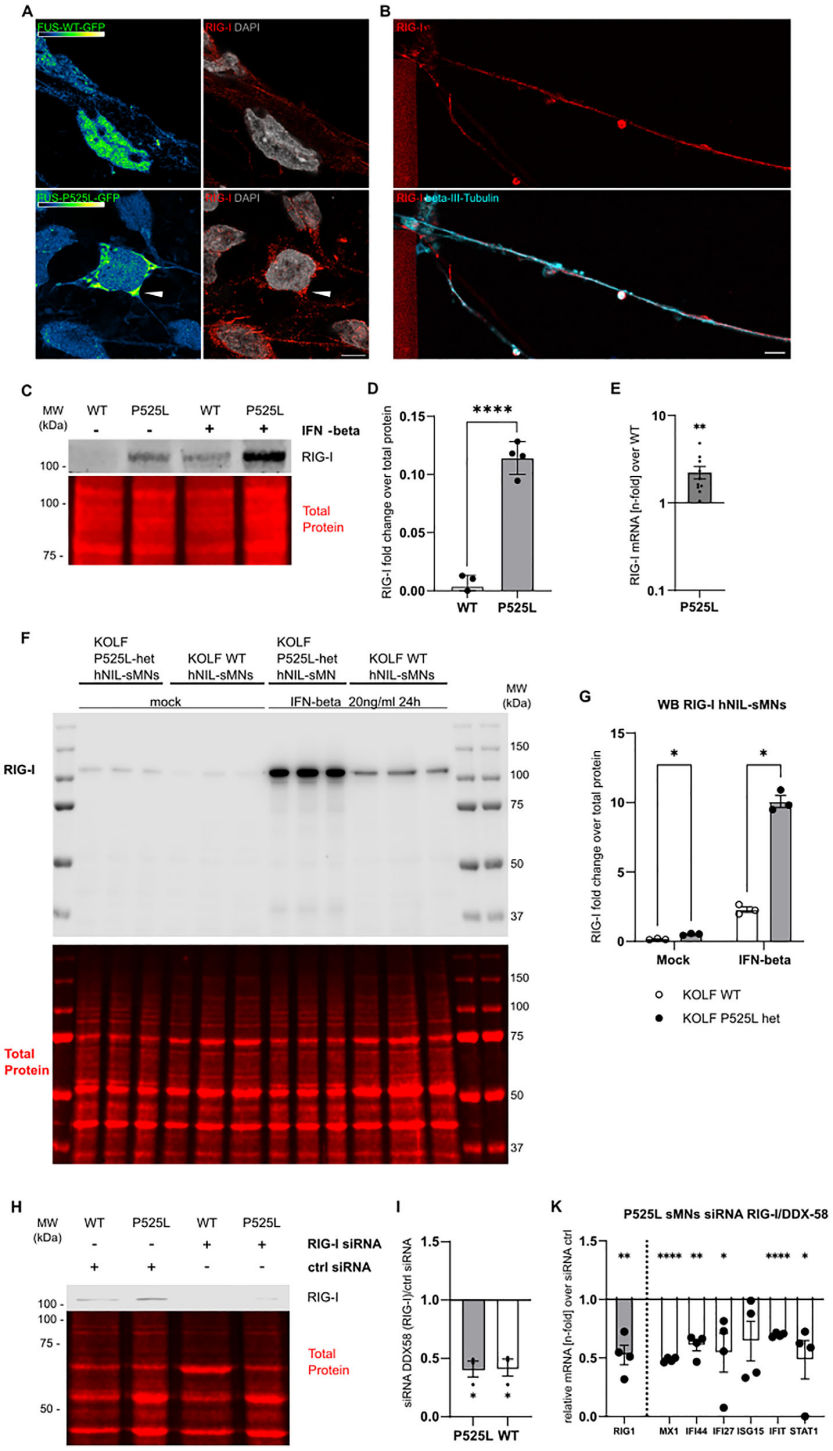
Expression of RIG‐I in FUS‐P525L sMNs. (A) IF panel of FUS^wt^ and FUS^mut^ sMNs counterstained for RIG‐I. Scale bar 10 µM. Note the enrichment in FUS‐GFP granules (white arrowhead). The GFP signal is depicted with the green‐blue‐fire LUT. (B): Example IF of RIG‐I indicating its presence in distal axons (mutant P525L sMN in this case). Scale bar 10 µM. (C) Cropped western blot scan of RIG‐I (100 kDa) in FUSwt and FUS‐P525L sMNs. IFN‐beta treatment (20 ng/mL 24 h) was performed as a positive control. (D) Quantification of (C) normalized to a total protein staining, n = 4, unpaired t‐test. Quantification was not done for the IFN‐beta treatment, as it only served as a control for antibody specificity. (E) Significantly higher RIG‐I mRNA expression was found in P525L‐sMN compared to control by RT‐qPCR, n = 10, unpaired t‐test. (F) WB scan including molecular weight markers for RIG‐I in KOLF WT and FUS‐P525L‐het hNIL‐sMN. IFN‐beta was used as a positive control. (G) Quantification of (F), unpaired t‐test, n = 3. (H) Cropped WB scan of RIG‐I for WT and FUS‐P525L sMNs treated with either ctrl‐siRNA or RIG‐I siRNA for 3d. Note the reduced signal of RIG‐I following RIG‐I siRNA treatment. (I) Quantification of (H), unpaired t‐test, n = 3. (K) RT‐qPCR in sMNs FUS‐P525L samples treated with siRNA against RIG‐I for 3d normalized to ctrl siRNA treated samples. Modest reduction of depicted ISGs including RIG‐I, Mann‐Whitney‐test for IFI27 and STAT1, otherwise the unpaired t‐test was used, n = 4 biological replicates.

Since we found significantly increased dsRNA in FUS^mut^ sMNs, we sought investigate further the role of the cytosolic dsRNA sensors MDA5 and RIG‐I in FUS^mut^ sMNs. Remarkably, we did not find a detectable signal for MDA5 on WB in either FUS^wt^ or FUS^mut^ sMNs. Since both sensors are ISGs themselves, stimulation of the pathway with IFN‐β was used as a positive control. A clear band of MDA5 was observed at the expected 140kDA (Suppl. Figure S2D). The same was true for RIG‐I, which showed a strong signal at 100 kDA after exposure to IFN‐β. Notably, however, a distinct band for RIG‐I was already detectable in FUS‐P525LsMNs under control conditions (Figure 3C,D; Supplementary Figure S5D). Similarly, this was reproducible when analyzing the KOLF P525L‐het hNIL‐sMN (Figure 3F,G). Interestingly, we found significantly higher signal for RIG‐I on the WB in the mutant hNIL‐sMN on baseline and under treatment with IFN‐beta.

Consistent with this, we also observed increased expression of RIG‐I in FUS‐P525L sMNs in immunofluorescence staining (Figure 3A) and significantly increased expression by qPCR (Figure 3G). Notably, RIG‐I was also highly expressed in the axons of motor neurons (Figure 3B).

NFX1‐type zinc finger–containing 1 (ZNFX1) is a highly conserved interferon‐stimulated dsRNA sensor able to initiate antiviral responses through MAVS [24]. While ZNFX1 is expressed in sMNs, there was no difference between FUS P525L and WT sMNs (Suppl. 5F).

We next asked whether RIG‐I activation was a cause of TBK1 stimulation and ISG activation or a consequence of being an ISG itself. To address this, we performed siRNA‐mediated knockdown of RIG‐I. Liposomal‐based siRNA treatment over 72 h resulted in an approximately 60% reduction of RIG‐I on the protein level (Figure 3H,I) and a robust decrease of RIG‐I mRNA in sMNs (Figure 3K). This was accompanied by a marked decrease in mRNA for the ISGs MX1, IFI27, IFIT, STAT1 and IFI44 (Figure 3K). Taken together, these data suggest that ISG activation in the FUS mutation is mediated by RIG‐I activation.

## 3 RIG‐I is Increased in Postmortem Spinal Motor Neurons of FUS Patients

**FIGURE 4:**
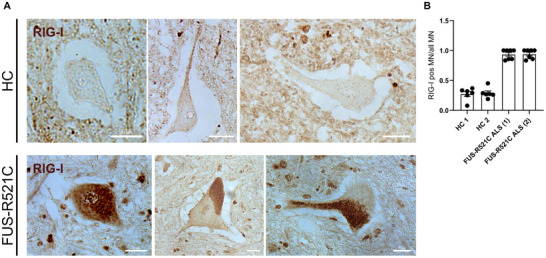
IHC for RIG‐I of spinal cord sections of a FUS‐ALS patient with a FUS‐R521C mutation and a healthy individual. (A) DAB‐IHC with an RIG‐I antibody. Representative examples of spinal cord sections of a healthy control individual (HC) and a FUS‐ALS patient(FUS‐R521C). Note the higher staining intensity in sMNs of the FUS‐ALS patient, which was particularly intense in axons. Scale bar = 20 µM. (B) Quantification of sMNs with RIG‐I inclusions or a strong cytosolic signal in two HC and two patients who had the same *FUS* mutation. Quantification was done by counting RIG‐I‐IHC positive and negative sMNs per whole spinal cord section. Interferon signaling induces axonal degeneration.

To further validate these in vitro findings, we analyzed RIG‐I expression by DAB‐IHC in spinal cord FFPE tissue sections of FUS‐ALS patients compared to healthy controls. While there was a faint cytoplasmic staining of RIG‐I‐I in healthy control (HC) α‐MNs, there was a markedly elevated DAB staining in case of FUS‐ALS patients (Figure 4A). We compared tissue from two unrelated patients with FUS‐ALS (R521C) with tissue from two individuals without neurodegenerative disorders (Suppl. Table S2). Quantification of spinal motoneuron count was done previously by us [14]. Both FUS patients had clear RIG‐I deposits in the majority of their sMN (Figure 4B).

**FIGURE 5:**
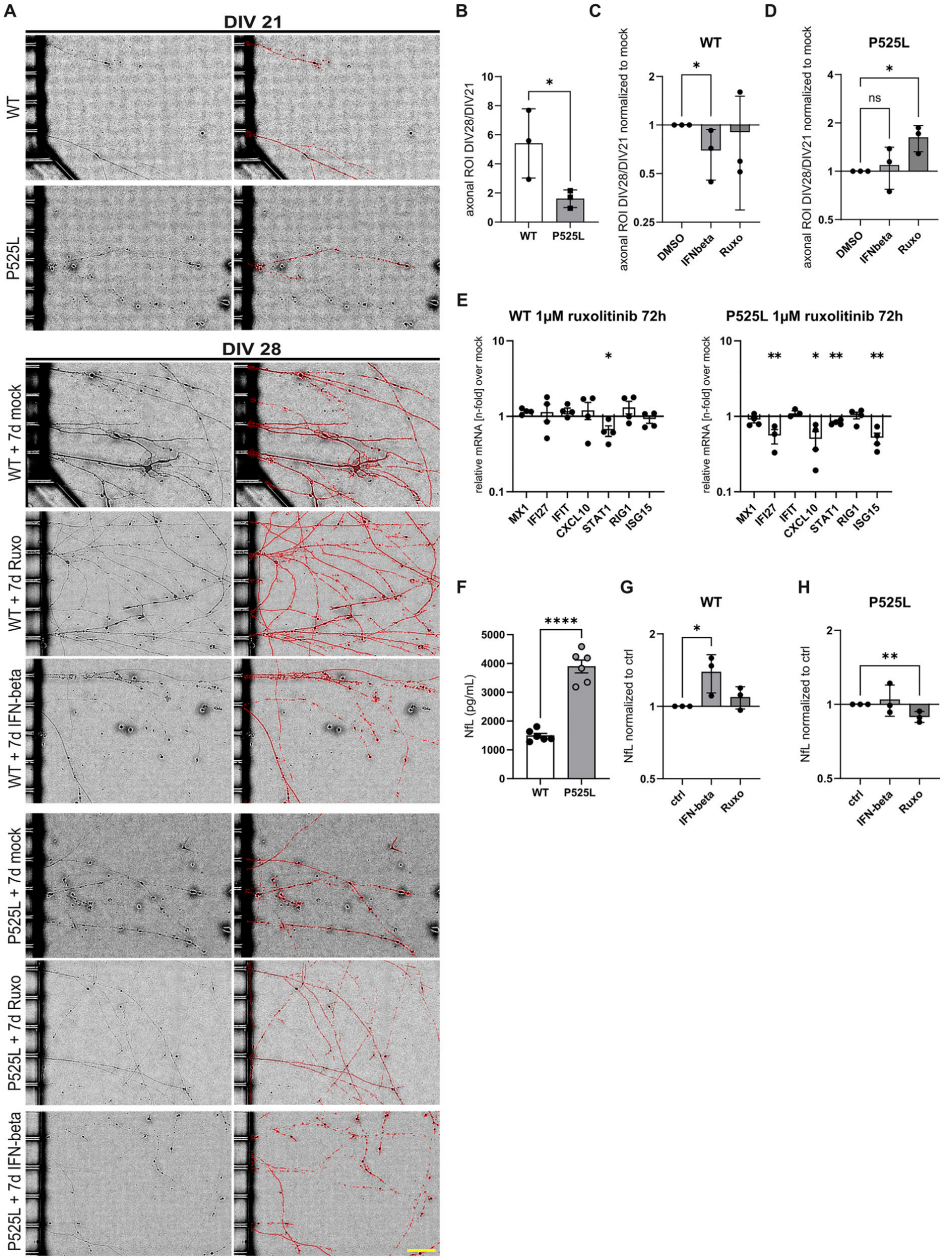
JAK Inhibition can partially reverse phenotypes in FUS sMNs. (A) Brightfield image panel (20×) showing the axonal compartment (DIV21) of the microfluidic chambers in which sMNs were cultured on the left (proximal) side. Subsequent imaging was performed after 7 days (DIV28) and image segmentation to detect axons was performed with Fiji as indicated by the red ROI area. Scale bar 50 µM, yellow, bottom left. (B) Quantification of the ROI area ratio (ROI DIV28/DIV21) indicating a significantly lower axonal growth in FUS‐P525L sMNs compared to control. N = 3, unpaired t‐test. (C,D) Axonal ROI ratio in FUS^WT^ (C) or FUS‐P525L (D) sMNs treated with IFN‐beta 20 ng or ruxolitinib 1 µM for 7d normalized to respective mock control, unpaired t‐test, n = 3, unpaired t‐test. (E) RT‐qPCR for a set of ISGs in FUS‐P525L sMNs treated with ruxolitinib for 72 h. Normalized to DMSO‐treated controls there was a partial reduction of the depicted ISGs, unpaired t‐test, n = 3. (F, G, H) Neurofilament light chain (NfL) was measured in medium supernatant by ELISA. Medium was collected after 72 h on DIV21 from FUS^wt^ and FUS‐P525L sMNs. (F) On baseline, there was a significantly higher level of NfL found in P525L‐sMNs compared to isogenic WT, unpaired t‐test, n = 6 biological replicates. (G,H) Cells were treated with either IFN‐beta 20 ng/mL or ruxolitinib 1 µM for 3d and NfL levels were normalized to the respective control condition (DMSO for ruxolitinib and PBS for IFN‐beta). N = 3 biological replicates, unpaired t‐test.

Considerable evidence has accumulated that DNA damage might be upstream of (axonal) neurodegeneration in different ALS models, including FUS [14, 17]. However, the link between the two remains to be elucidated. Previous reports have demonstrated functional axonal deterioration and neurodegenerative phenotypes after exposure of neurons to TNF‐α or IFN‐β [18, 19]. In light of these previous findings, we asked whether IFN1 activation might be responsible for axonal damage and neurodegeneration. First, we found a robust upregulation of IFN‐β in KOLF P525L hNIL‐sMN compared to its isogenic control (Suppl. Figure S2E). We next exposed mature motor neurons to IFN‐β for 7 days and assessed the axonal growth area by brightfield imaging before and after treatment (Figure 5A–D). For selective axonal analysis, we used microfluidic chambers, which allow the cultivation of neuronal somata spatially separated from their outgrowing axons, as previously shown [14]. While WT axons showed marked growth over the 7 days as measured by the area ratio normalised to the area just before treatment, the growth of FUS^mut^ axons was significantly reduced (Figure 5A,B). Treatment with 20 ng/mL IFN‐β reduced axonal area in WT neurons but had no additional effect on FUS^mut^ sMNs axons (Figure 5D). To validate this structural phenotype, we measured neurofilament light chain (NfL) in the media supernatant as a surrogate for axonal damage and a well‐established diagnostic and prognostic biomarker in the clinic [25]. Medium of mature sMNs was collected after 3 days of IFN‐β or mock treatment and measured using ELLA. First, we observed a strikingly higher NfL amount in FUS^mut^ vs. FUS^wt^ sMNs under baseline conditions (Figure 5F). Importantly, IFN‐β led to a significant NfL increase in FUS^wt^ sMNs, while mutant sMNs did not change. These results suggest that IFN signaling is harmful for sMNs, leading to neurodegeneration, and even more, that the IFN‐1 signaling cascade might be one mediator of the observed axon degeneration in FUS‐ALS sMNs.

### 3.1 Axonal Damage Can be Mitigated by JAK Inhibition

RIG‐I/TBK1/IRF3 activates IFN‐1, which upon release can activate its receptor in an autocrine or paracrine fashion. Binding of IFN‐1 to its receptor subsequently activates the JAK‐STAT pathway, for which FDA‐approved inhibitors are available. To address whether the JAK‐STAT pathway is activated, we performed a pSTAT1 western blot. Because STAT1 is a phosphorylation target of JAK, the detection of pSTAT1 would indicate a pathway stimulation. In fact, we could find a significant signal of pSTAT1 in KOLF P525L‐het hNIL‐sMN compared to its isogenic KOLF WT control (Suppl Figure S3D,E,H,I). Interestingly, treatment with IFN‐beta, which serves as a strong positive control for the pSTAT1 signal, also resulted in a significantly stronger pSTAT1 response in the KOLF FUS P525Lhet hNIL‐sMN compared to its isogenic WT. After the establishment of an involvement of the JAK‐STAT pathway, we exposed mature sMNs to the selective JAK inhibitor ruxolitinib [26] for 7 days and assessed the axonal growth area by brightfield imaging before and after treatment (Figure 5A–D). While WT axons showed no change in their outgrowth behavior in the presence of ruxolitinib, FUS^mut^ axonal growth could be significantly restored by treatment with 1 µM ruxolitinib for 7 days (Figure 5D). Fitting to this, Ruxolitinib also significantly reduced ISGs in FUS^mut^ sMNs, but not in FUS^wt^ sMNs (Figure 5E). Medium supernatents of mature sMNs was collected after 3 days of ruxolitinib or sham treatment and measured using ELLA. Ruxolitinib resulted in a significant reduction of NfL in FUS^mut^ sMNs, which was not seen in FUS^wt^ sMNs (Figure 5G,H). These results support the idea of an existing activation of the IFN pathway in FUS^mut^ sMNs, but not in FUS^wt^ sMNs, which can be alleviated by JAK inhibition.

### 3.2 Upregulated Mitochondrial Transcription in Axonal FUS‐P525‐sMNs Samples

**FIGURE 6:**
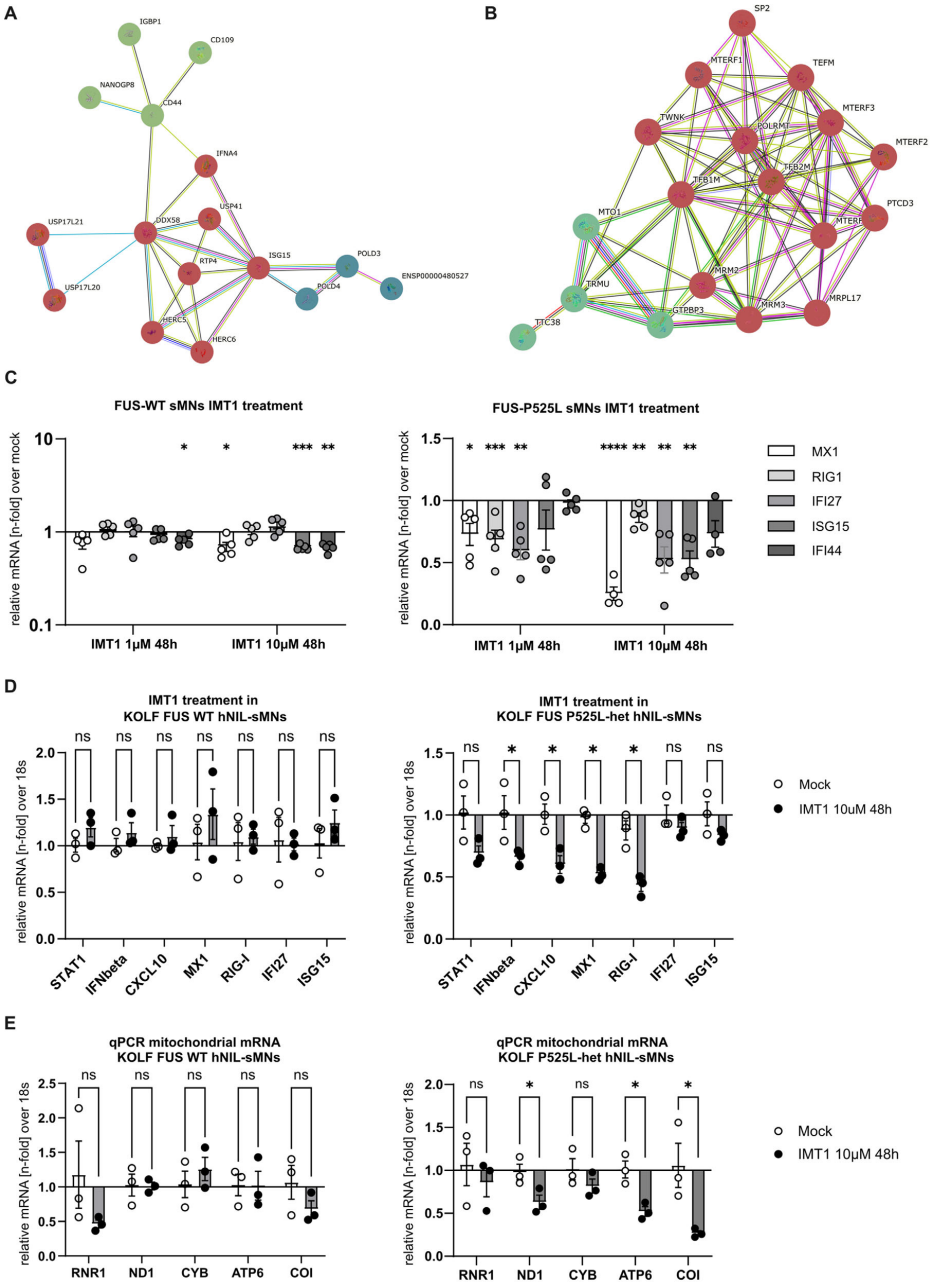
Axonal RNA sequencing reveals upregulated mitochondrial transcription in axonal FUS‐P525L sMNs samples. The STRING PPI network based on transcriptome analysis from RNA‐seq data of axonal sMNs samples. (A) Cluster analysis indicates significant upregulation of innate immune activation with RIG‐I in the center and (B) mitochondrial transcription pathway enrichment. In the PPI network, each node represents a protein/DEG, and each edge represents an interaction. The basic settings in this network are as follows: The network type is “full network” (the edges indicate both functional and physical protein associations) and the line color indicates the type of interaction evidence; the active interaction sources include “text mining, experiments, databases, co‐expression, neighborhood, gene fusion, and co‐occurrence.” The minimum required interaction score was set as “medium confidence” (confidence score ≥ 0.400), and the limitation of the max number of interactors in each cluster was set as “10 interactors.” The interacting proteins have been clustered (Markov cluster algorithm (MCL) clustering, inflation = 3) based on their functions and associations to select the most significant functional clusters. Coloring is based on STRING default color paraments. (C) Dose‐dependent drop of ISGs measured by qPCR in FUS‐P525L sMNs after treatment with the non‐competitive, human mitochondrial RNA polymerase (POLRMT) inhibitor IMT1. In comparison there was only a very modest reduction of two ISGs in the WT, one‐Way ANOVA, Tukey's post hoc, n = 5 biological replicates. (D) ISG measurement by qPCR in KOLF WT and P525‐het hNIL‐sMN after IMT1 treatment (10 µM 48 h), indicating a significant reduction of several ISGs, including IFN‐beta itself, in the FUS‐P525L hNIL‐sMN, unpaired t‐test, n = 3). (E) qPCR for several mitochondrial mRNA transcripts after IMT1 treatment (10 µM 48 h) in KOLF WT and P525‐het hNIL‐sMN serving as a positive control for the IMT1 effect of mitochondrial polymerase inhibition. While there was only a trend in the KOLF WT, several mitochondrial mRNA transcripts were significantly lower expressed in the KOLF FUS‐P525L het hNIL‐sMN after treatment with IMT1 compared to mock (DMSO), unpaired t‐test, n = 3.

Having shown increased cell‐autonomous IFN1 signalling in sMNs of FUS‐ALS patients responsible for axonal degeneration, together with prominent axonal RIG‐I expression (Figure 3), we wondered whether the source of increased extramitochondrial dsRNA was indeed increased mitochondrial transcription. To this end, we used our previously described RNA sequencing (RNA‐seq) dataset in which we performed transcriptomics of somatodendritic vs. axonal compartments of FUS^wt^ vs. FUS^mut^ sMNs using microfluidic devices [27]. To identify possible protein‐protein interaction networks, we performed STRING analysis on the axon‐specific differentially expressed genes (DEGs) between FUS^wt^ and FUS^mut^ datasets. First, we found a protein‐protein interaction network of axonally enriched genes of mitochondrial/metabolic functions (Figure 6A). Notably, using this unbiased sequencing approach, we also identified an axonally upregulated gene cluster involved in RIG‐I‐based innate immune activation (Figure 6B). Mitochondrial transcription is carried out by the single‐subunit mitochondrial RNA polymerase (POLRMT). We therefore wanted to investigate whether increased mitochondrial transcription could be the source of ISG activation. We used the non‐competitive human mitochondrial RNA polymerase (POLRMT) inhibitor IMT1 [28]. IMT1 inhibition was shown previously to lower dsRNA in human cells [29]. This led to a dose‐dependent drop of ISGs in FUS‐P525L sMNs, but only very moderately in the FUS‐WT sMN (Figure 6C), which could also be reproduced in KOLF P525L‐het hNIL‐SMN (6D). To validate the specificity of IMT1, we also checked for the reduction of mitochondrial mRNA transcripts after IMT1 treatment and found a significant decrease of several mitochondrial‐specific transcripts (Figure 6E). Finally, we also asked if IMT1 can, in fact, reduce the J2‐dsRNA signal. Treatment with 10 µM IMT1 over 48 h caused a modest reduction of J2 in WT sMN, and markedly diminished J2 signal in FUS P525L sMN (Suppl. Figure S6L).

These data suggest that an increased mitochondrial transcription in FUS^mut^sMNs led to excessive dsRNA production, which triggers the innate immune system via RIG‐I.

### 3.3 IFN1 Activation Is Found in Peripheral Blood Samples of FUS‐ALS Patients

**FIGURE 7:**
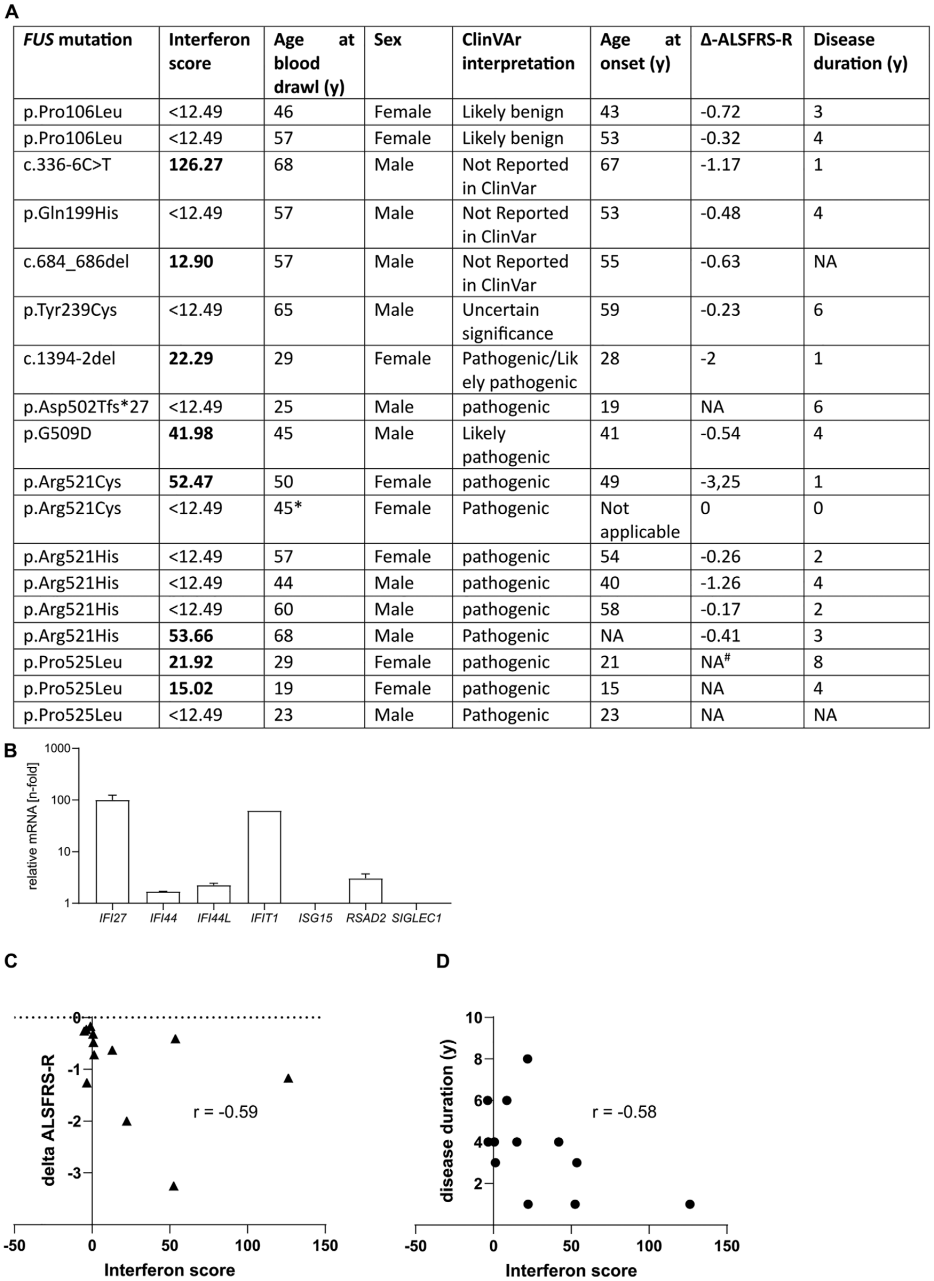
IFN score assessment in blood samples of FUS‐ALS patients. (A) Analysis of the interferon stimulated gene response in 18 ALS patients with mutations in *FUS* including assessment of clinical parameters. Seven mRNA transcripts were analyzed as indicated in (B) with qPCR normalized to HPRT and GAPDH. The interferon signature was calculated as described before [31]. An IFN score of 12.49 indicates the cut‐off for significant results and is calculated as the median of 10 healthy controls plus 2.5×SD. Any IFN score higher than 12.49 is considered increased. (B) Example of one blood ISG transcription measurement in a FUS‐ALS patient with a FUS c.336‐6C>T mutation, which has not been reported previously. (C,D) A Significant correlations were found between the Δ‐ALSFRS‐R and the IFN score (Spearman r = −0.5879, p = 0.038, one‐tailed t‐test),, but also to the disease duration (Spearman r = −0.58, p = 0.04, one‐tailed t‐test). * pre‐symptomatic patient with pure fasciculations and signs of denervation on EMG. ^#^ patient was locked in for several years at the time of the blood drawl.

Our previous results showed a significant neuron‐specific upregulation of IFN1 signaling in various patient‐derived spinal motor neurons and post‐mortem spinal cord from FUS‐ALS patients. However, we also wondered whether there is also a systemic activation of type I interferon signaling. We therefore analyzed ISG upregulation using the Interferon Signature Score (ISS), which combines a number of ISG measured by qPCR in peripheral blood samples (Figure 7A,B). This assessment of IFN1 upregulation is widely used in rheumatology and correlates with disease activity in inflammatory diseases such as systemic lupus erythematosus [30].

We thus investigated 18 FUS‐ALS patients with different *FUS* mutations for an interferon signature (Figure 7). We performed RT‐qPCR to calculate the interferon score, which is considered to be increased if greater than 12.49, which is the median + twofold SD of a healthy control group. Interestingly, eight out of 18 FUS‐ALS patients showed an increased interferon score (Figure 7A). Notably, only two patients with an N‐terminal mutation had an elevated interferon score, whereas 50% of C‐terminal mutation carriers had an elevated score. We further correlated the interferon score with several clinical variables. We found a significant correlation of the ISS with the delta‐ALSFRS‐R, which is a measure of disease progression (Spearman r = −0.59, p = 0.024, Figure 7C) and disease duration (Spearman r = −0.58, p = 0.04, Figure 7D), but not with age at onset (Spearman r = −0.49, p = 0.08).

## 4 Discussion

The vast majority of clinical trials in ALS still fail by phase 3. One reason may be the heterogeneous nature of ALS and the lack of pathophysiological‐driven biomarkers for patient stratification. IFN‐1 pathway activation, an emerging field in ALS research, may provide an opportunity to fill this gap. It is also of interest because it culminates in a pathway for which FDA and EMA approved drugs are available, irrespective of the different upstream mechanisms involved in its activation. Accordingly, IFN‐1 activation has been reported in various genetic ALS model systems and patients, mainly involving cGAS STING‐dependent pathways, although it appears that in ALS a plethora of upstream mechanisms putatively exist to trigger these pathways depending on specific mutations [4, 11, 12, 13, 32].

Since the pathophysiology of FUS‐ALS includes impairment of DNA damage repair as well as mitochondrial damage, we hypothesized that one of these processes might be involved in aberrant IFN1 activation. Using isogenic and non‐isogenic iPSC‐derived sMNs with different differentiation protocols as well as post‐mortem spinal cord tissue, we were able to demonstrate an IFN1 pathway activation in FUS‐ALS, which was dependent on RIG‐I activation. RIG‐I was increased in sMNs axons of FUS^mut^ iPSCs as well as in α‐MNs of FUS‐ALS post‐mortem spinal cord, presumably through increased mitochondrial transcription. Notably, IFN signaling induced axonal degeneration in sMNs. Finally, ISG activation was found in the peripheral blood of half of FUS‐ALS patients and correlated with disease aggressiveness and duration. Janus kinase inhibition by the FDA‐approved drug ruxolitinib reversed not only ISG signaling but also axonal damage and neurofilament release of diseased neurons, supporting it as a potential candidate for a biomarker‐driven target for (FUS‐)ALS.

While most studies of IFN‐1 activation have been conducted in non‐neuronal cells, including immune cells, only a few recent reports have implicated neuronal STING activation in ALS and FTD, including analysis of post‐mortem cortical and spinal cord [2, 4, 13]. The in vivo data remarkably showed that this STING activation was most prominent in the most affected neuronal subpopulations in ALS, namely cortical layer V and spinal α‐sMNs [13]. Furthermore, pathway components were found intra‐neuronally, suggesting the cell‐autonomous nature of activation with DNA damage as the main contributor. However, FUS‐ALS was not included in this study. Interestingly, our results show that cell‐autonomous IFN‐1 activation is also present in FUS‐ALS sMNs, although not primarily induced by nuclear DNA damage and subsequent STING activation. This was supported by the lack of IFN‐1 activation after DNA damage induction and the lack of efficacy of the STING inhibitor H151 in reducing ISGs in the FUS^mut^ sMNs.

In contrast, we were able to delineate the importance of RIG‐I activation in FUS^mut^ sMNs to trigger the innate immune response. Consistent with the role of RIG‐I in sensing dsRNA species, we found abundant dsRNA in the cytosol of FUS^mut^ sMNs, where RIG‐I resides. Consistent with this, our RNA‐seq data indicated an up‐regulation of a network centered around RIG‐I/DDX58. Furthermore, axonal RNA‐seq revealed an upregulated mitochondrial RNA transcription pathway that could promote the accumulation of immunogenic dsRNA in FUS‐P525L sMNs. Notably, pharmacological inhibition of mitochondrial transcription with IMT1 [28] reduced ISG expression in FUS‐P525L sMNs, further supporting the mechanism of dsRNA‐mediated innate immune stimulation in our FUS sMNs model (Figure 6C,D). However, future studies are needed to elucidate the source and fate of immunogenic dsRNA in sMNs in more detail. One could speculate on mechanisms that prevent aberrant innate immune activation by dsRNA in neurons. One such mechanism could be the integration of dsRNA into stress granules, the absence of which has been reported to lead to hyperactivation of type I interferon [33, 34]. However, the binding of viral RNA and dsRNA to G3BP1 has been reported to trigger an IFN1 response mediated by RIG‐I [35]. Interestingly, stress granule assembly was reported to be required for IRF3‐mediated IFN production, but not IFN signaling or proinflammatory cytokine induction [36]. In FUS‐ALS, cytoplasmic mislocalization is reported to induce stress granules and these show aberrant dynamics [37], thus the disturbed stress granule function might indeed trigger IFN1 response activation. Furthermore, compensatory mechanisms such as heat shock factor expression or integrated stress response activation are already increased under baseline conditions in FUS‐ALS [37], thus any additional event affecting stress granules might be sufficient to neutralize stress granules's protective effect. Further work is thus needed to clarify the role of stress granules in dsRNA‐RIG‐I mediated IFN1 activation and particularly in FUS‐ALS.

Given reports of both physiological functions of FUS in mitochondrial DNA repair [38] and pathological accumulation of mutant FUS leading to HSP60 dysfunction [39], it will be important to investigate the role of FUS with a focus on mitochondrial dsRNA maintenance, as both loss‐of‐function and gain‐of‐function mechanisms could apply. Mechanistically, our experiments support a RIG‐I‐driven sensing of the abundant cytosolic dsRNA in FUS^mut^ sMNs and subsequent innate immune stimulation. In contrast, we did not find a meaningful signal for MDA5, another established dsRNA sensing enzyme upstream of TBK1 activation. We also didn't detect differences in ZNFX1 expression (Suppl. Figure S5F), which is an interferon‐stimulated dsRNA sensor able to initiate antiviral responses through MAVS. Our data also revealed enrichment of RIG‐I in post‐mortem sMNs from FUS patients, validating these findings. Importantly, accumulation of endogenous RNAs as a result of TDP‐43 loss of function has previously been shown to cause RIG‐I‐dependent necrotic cell death [32]. Post‐mortem evidence for neuronal cell‐autonomous innate immune activation already exists for both, sporadic and genetic ALS models [13, 40, 41], but highlighted the role of STING signaling in this. This has been experimentally attributed to upstream DNA damage events leading to STING activation in vitro. However, this does not contradict our data, as STING itself has been shown to be an ISG [42]. Therefore, the strong post‐mortem signal in sMNs in the presented singular FUS‐ALS case [13] could be due to primary RIG‐I signaling and secondary STING stimulation as an ISG, which could also support our finding of slightly, but not significantly, increased STING on the WB (Figure 1H). Consistent with this, the STING inhibitor H151 failed to reduce ISGs in FUS^mut^ sMNs in our experiments (Figure 1M–O). This would support the difference between a possible upregulation by innate immune stimulation and the primary activation by cGAS, which should be blocked by H151.

Regardless of the primary mechanism, the common downstream signaling pathway is a promising drug target. Our data and others [10] suggest that drugs such as ruxolitinib, whose JAK inhibitory mode of action covers a broad spectrum of IFN1 signaling effects including STING, MDA5 and RIG‐I mediated ones, could not only reduce ISG and NfL levels in FUS^mut^ sMNs but also attenuate axonal degeneration in our cell model.

While both, impairment of DNA damage repair and axonal mitochondrial trafficking deficits and depolarization have been reported as central phenotypes in FUS‐ALS, their connection so far remained enigmatic [14, 16, 43]. While DNA damage induction was sufficient to phenocopy FUS‐ALS mitochondrial deficits [14], it did not induce an IFN1 response. However, IFN‐β treatment was indeed able to induce axon degeneration (Figure 5). Mutant FUS was reported to localize to mitochondria, being involved in mitochondrial DNA damage repair [38]. Since we and others recently showed that increased DNA damage leads to activation of DNA‐PK and this results in further increase of cytoplasmic mislocalization of FUS putatively due to its phosphorylation closing a vicious cycle [14, 44], we speculate that the increased cytoplasmic FUS increasingly culminates in mitochondria and stress granules, both of which are involved in RIG‐I mediated IFN1 activation. The latter then becomes suicidal, including the observed axon degeneration. However, this hypothesis requires further investigation.

A limitation of the study arises from the relatively small number of FUS‐ALS patients measured. Of note, however, FUS‐ALS is a rare condition and particularly rapidly progressive, thus 18 patients already consists of a meaningful sample. Nevertheless, future studies are needed to validate the blood ISG findings in larger cohorts. The same accounts for the post mortem findings. Another limitation of the study is that no CSF was available to analyze the IFN1 response in the CSF of FUS‐ALS patients. Of note, however, ISG activation by the qPCR would be difficult to assess in CSF due to the low cell numbers in non‐inflammatory neurodegenerative diseases. Alternatively, ISGs such as CCL2 have been reported to be increased at the protein level in ALS CSF, which was not always seen in serum, so sensitivity may be even higher when analysing CSF [45]. In addition, the detailed source of RIG‐I activation requires further investigation, including the question of the role of FUS loss or gain of function in this context. In addition, the role of increased cytosolic DNA and the cGAS‐STING pathway and its putative crosstalk with the RIG‐I pathway in FUS‐ALS is of further interest as well. Finally, future studies are needed to systematically and differentially assess cell type and body compartment differences in IFN1 activation in ALS and correlations with neuropathology‐driven biomarkers such as cryptic peptides [46] or extracellular vesicle TDP43 [47].

## 5 Methods

### 5.1 Sex as a Biological Variable

ALS is a rare disease, and genetic forms are even rarer. It is thus difficult to stratify for sex. We tried to include cell lines, post‐mortem and patient blood from both sexes.

Cell lines: We included isogenic lines as well as non‐isogenic line which had been sex‐ and aged‐matched, thus both sexes were included.

Post mortem: We only got access to post‐mortem of female FUS patients, and matched them to female control cases. Since there is no evidence yet that the pathophysiology differs between males and females in FUS‐ALS, we do not expect that there are differences by sex.

Patients: ISG measurements in patient blood were performed in males and females.

### 5.2 Cell Culture

iPSC‐derived sMNs culture was accomplished as demonstrated previously [14, 37]. The iPSC generation and CRISPR‐Cas9n creation of the isogenic pair of lines of hiPSC and NPC of WT FUS‐eGFP and P525L FUS‐eGFP is described therein including the generation of the other cell lines carrying FUS mutations (R521C, R521L, R495Qfs*527) and two healthy, non‐isogenic control lines. Note, that the non‐isogenic FUS^mut^ cell lines were only used for experiments demonstrated in Figure 1. NPC culture and differentiation was performed via a modified protocol by Reinhardt et al. [48]. Briefly, NPC were maintained in the basic medium (DMEM‐F12/Neurobasal 50:50 medium with N2 Supplement (1:200), B27 Supplement without vitamin A (1:100), penicillin/streptomycin/glutamine (1%), supplemented with Chiron‐99021 (3 µM), Ascorbic acid (150 µM) and Purmorphamine (0.5 µM) on dishes coated with Matrigel. For coating, Matrigel was diluted 1:100 in Knock‐Out DMEM and added to dishes, followed by the incubation at 37°C for at least 1 h. NPC were split after reaching 70–80% confluence at a 1:10 ratio using Accutase for 10 min at 37°C. Induction of sMNs differentiation was performed by adding basic medium supplemented with BDNF (1 ng/mL), Ascorbic acid (200 µM), Retinoic acid (1 µM), GDNF (1 ng/mL) and Purmorphamine (0.5 µM) to freshly split NPCs and maintained for 5 days with the medium changed every other day. For the final maturation, the medium was changed on day 6 to the basic medium supplemented with DBcAMP (100 µM), BDNF (2 ng/mL), Ascorbic acid (200 µM), TGFβ‐3 (1 ng/mL) and GDNF (2 ng/mL). On day 9, cells were split onto the dishes coated with Poly‐L‐ornithine and laminin and maintained for at least 21 days (DIV21) before they were used for the final analysis. In case of the brightfield experiments shown in Figure 5, cells were kept for an additional 7 days in culture (DIV28). Furthermore, in this experiment cells were grown in microfluidic chambers (MFC) allowing selective axonal assessments. MFC culture for iPSC‐derived sMNs was demonstrated previously by us [14].

The KOLF iPSC were created by the iNDI consortium [49] and were available through Jax (https://www.jax.org/jax‐mice‐and‐services/ipsc). Differentiation of sMN with an hNIL piggyBac construct was performed as previously reported [20]. Human induced pluripotent stem cells (iPSCs) were engineered to stably express a doxycycline‐inducible hNIL transcription factor cassette (NGN2, ISL1, LHX3) using a piggyBac transposon system. Briefly, iPSCs were co‐transfected with the piggyBac‐hNIL construct and a piggyBac transposase helper plasmid, followed by puromycin selection (10 µg/mL) to isolate stable integrants. Selected clones were expanded under feeder‐free conditions on Matrigel in mTeSR medium and maintained for at least three passages prior to differentiation.

For induction, iPSCs were dissociated to single cells and replated onto Matrigel‐coated plates in mTeSR medium containing CET (1000× stock, Chroman1 (50uM, MedChem Express HY‐15392), Emricasan (5 mM, Selleckchem S7775), Trans‐ISRIB (0.7 mM, Tocris 5284)). The following day (Day 1), doxycycline (2 µg/mL) was added to initiate hNIL expression and neuronal conversion. From Day 1 onward, the medium was switched to a neuronal differentiation medium (DMEM/F12, supplemented with N2 (100×), GlutaMAX (100×), non‐essential amino acids (100×) and Pen‐Strep (100×), Compound E (1 mg/mL, Calbiochem 565790‐500UG, resuspend in 1:1 ethanol:DMSO). Cells were gently replated with Accutase on poly‐L‐ornithine/laminin‐coated plates at Day 3 to promote neurite outgrowth and maintained under continuous doxycycline induction and CET was added to the medium. On day 4, the medium was changed to neuronal long‐term medium without doxycycline (Neuralbasal (Lifetech 21103049), Non‐essential amino acids (100×) (Corning 25‐025‐CI), Glutamax (100×) (thermo 35050061), N2 supplement (gibco 17502‐048), B‐ME (25 mM) (Sigma M6250‐100ML), Pen/Strep (100×) (Lifetech 15070‐063). Differentiated spinal motor neurons were used for experiments on Day 10 after initiation of doxycycline treatment. As a positive control for the GFAP IF, ScienCell primary astrocytes (#1800) were added to the cell culture on Day 6 after removal of aphidicolin.

The cells were regularly tested for mycoplasma contamination.

### 5.3 Patient Characteristics

Cell lines carrying a clinically more benign phenotype (R521C, L) or more severe (R495QfsX527, P525L) FUS mutation were included in this study and systematically compared to two control iPSC lines from healthy volunteers or to an isogenic line. All procedures were in accordance with the Helsinki Convention and approved by the Ethical Committee of the University Medical Center Rostock (A2019‐0134) and Dresden (EK45022009) Patients and controls gave written consent before skin biopsy or blood sampling for ISG measurement. In total, 18 patients were identified with a mean age of 47 at blood drawl. Due to the rareness of the disease, all patients with a heterozygous mutation in FUS were included independent of their disease stage. The female‐male ratio was 0.44. The delta‐ALSRFS‐R was calculated as the ratio of the maximum ALSFRS‐R (48) minus the ALSFRS‐R at the point of blood drawl divided through the estimated number of months since occurrence of first ALS symptoms.

### 5.4 Treatments and siRNA

Etoposide (Sigma‐Aldrich E1383) was dissolved in DMSO to obtain a 10 mM stock. ABT‐888 (PARP1 inhibitor, Santa Cruz Biotechnology sc‐202901) was dissolved in DMSO to obtain a 20 mg/mL stock. Recombinant human interferon‐beta (PeproTech, 300–02BC) and recombinant human TNF‐alpha (PeprotTech, 300–01A) were resuspended to a final stock of 20 µg/mL in sterile aqua dest. Ruxolitinib was obtained from MedChemExpress (HY‐50856) and dissolved in DMSO for a 10 mM stock. The STING inhibitor H151 was obtained from Tocris (6675) and dissolved in DMSO to a stock of 5 mM. Thapsigargin was from Sigma (T9033), dissolved in DMSO, and kept at ‐80°C. IMT1 was purchased from MedChemExpress (HY‐134539) and dissolved in DMSO. siRNA Silencer Select was from Invitrogen (DDX58/RIG‐I 4392420; Negative Control 4390843). Silencer Select siRNA was dissolved in nuclease‐free water according to the manufacturer's instructions. Transfection was done using FUSE‐it siRNA (beniag GmbH) according to the manufacturer's instructions. In brief, the master mix was produced according to the manufacturer's instructions. The sMNs were then incubated for 20 min in an incubator at 37°. RNA or protein isolation was performed after 72 h.

### 5.5 Microscopy and Immunofluorescence

Cells were fixed in vitro with ice‐cold 4% paraformaldehyde for 15 min, then permeabilized in 0.2% Triton X‐100 for 10 min. Afterward, cells were washed three times with PBS for 5 min and blocked in Pierce Protein‐free blocking buffer (37572; Thermo Fisher) for 1 h at room temperature. The following primary antibodies were diluted in Pierce Protein‐free blocking buffer and incubated overnight at 4°C: Anti‐RIG‐I (1:500, clone 1C3, MABF297 Sigma‐Aldrich), anti‐dsRNA J2 (1:500, SCICONS 10010200); IgM‐anti‐DNA 30‐10‐10 (1:500, Progen 690014), anti‐TOM20 (1:1000, Invitrogen, MA5‐34964), anti‐HSP60 (1:1000; ab46798; Abcam), anti‐Tuj1 (1:500, aveslabs), anti‐Isl1/2 (DSHB, 39.4D5), anti‐GFAP (InvitroGen 13–0300),. Afterward, cells were washed thrice in PBS for 5 min, secondary antibodies were diluted 1:1000 in blocking buffer and incubated for 1 h at room temperature, on a shaker. Following three more washing steps with PBS for 5 min, cells were mounted in DAPI Fluoromount‐G mounting medium (0100‐20; Southern Biotechnology). Images were acquired on a Zeiss inverted AxioObserver.Z1 microscope with LSM 900 module and high‐resolution Airyscan 2 module, using a 63 × 1.4 NA plan apochromat objective.

### 5.6 Image Analysis With Fiji

CZI images were imported and edited in FIJI (2.15.1). The Labkit plugin (15) was applied for image segmentation to generate defined classifiers for, for example, the fluorescence signal of dsRNA. From this, ROIs were created for each signal of interest. Using logical operators the nuclear area was removed and the area proportion of dsRNA inside and outside of the HSP60+ ROI was quantified. This was normalized to the total cell area measured with a background GFP classifier.

For MFC brightfield quantification, the total area of the right MFC compartment was captured with a 20× objective and repeated imaging with an image overlay of 10%. Image fusion was accomplished with a Zeiss ZEN Blue built‐in processing algorithm. Axonal segmentation for ROI recognition was performed with the Labkit plugin to capture the entire axonal growth area within the dish.

### 5.7 Statistics

Statistical testing was performed in GraphPad Prism 10. Statistical tests are described in the figure captions. Asterisks indicate significance in figures as a result of statistical testing: * *p* < 0.05, ** *p* < 0.01, ***p < 0.001, **** *p* < 0.0001.

### 5.8 Western Blot

Cell pellets were scraped from the dish and snap‐frozen. Afterward, they were lysed in RIPA buffer containing EDTA‐free protease inhibitor cocktail (04693132001; Roche) and phosphatase‐inhibitor (Roche PhosSTOP EASYpack 04906837001) for 30 min on ice. The lysate was incubated with Roti‐Load (Carl Roth, K929.1) and heated for 5 min at 96°C. A total of 30 µg of protein was loaded on precast polyacrylamide gels (*Bio‐Rad* 4%–15% Criterion TGX Stain‐Free Precast Gels, 18 Well Comb, 30 µL, 1.0 mm, 5678084) for gel electrophoresis. Proteins were blotted on 0.2‐µm nitrocellulose membrane (Trans‐Blot Turbo Midi 0.2 µm Nitrocellulose Transfer Packs 1704159; BioRad), using a Trans‐blot Turbo transfer system (22 V, 7 min; Bio‐Rad). Total protein was stained with REVERT Total Protein Stain (Li‐Cor: 92611011) according to the manufacturer's instructions. Membrane blocking was done in 5% non‐fat milk in TBS‐T (T145.3; in TBS; Roth) for 1 h at room temperature. Primary antibodies were diluted in blocking solution and kept on the membrane for 1 h at room temperature if not stated otherwise. For WB the following primary antibodies were used: rabbit anti‐MDA‐5 (Cell Signaling, D74E4); rabbit anti‐TBK1 (Invitrogen, PA5‐17478), rabbit anti‐RIG‐I (D14G6,Cell Signaling, #3743), rabbit anti‐phospho‐TBK1 (Ser172, D52C2, Cell Signaling, #5483), mouse anti‐IRF3 (Invitrogen, 14‐9947‐82 eBioscience), rabbit anti‐STING (Novus, NBP2‐24683), rabbit anti‐p65 (C22B4, Cell Signaling, #4764), rabbit anti‐phospho‐Ser536‐p65 (93H1, Cell Signaling, #3033), anti pSTAT1 Y701 (Cell Signaling, #9167S). Secondary antibodies (anti‐rabbit IRDye 800CW abcam Ab216773, anti‐mouse IRDye 800CW abcam ab216772, anti‐rabbit IRDye 680RD abcam ab216777, anti‐mouse IRDye 680RD abcam ab216776) were incubated in TBS‐T overnight at 4°C. Images were acquired on a Li‐COR ODYSSEY XF analyzer. Image analysis was performed with the Empiria Studio 3.0 software (Li‐COR).

### 5.9 Quantitative RT‐PCR

RNA was extracted from isolation cell pellets and isolated with the Quick‐RNA Miniprep Plus Kit (Zymo Research R1057T) according to the manufacturer's instructions. cDNA was generated with the High‐Capacity cDNA Reverse Transcription Kit (ThermoFisher 4368814) using 200 ng of isolated RNA according to the manufacturer's instructions. Gene expression was determined by quantitative rt‐PCR using the FastStart Essential DNA Green Master kit (06402712001) in a LightCycler 480 II (Roche) and normalized to the mean of the 18s and HPRT expression. The following primer sequences were used in this work: 18s fwd CGTAGTTCCGACCATAAACGATGCC and rev GTGGTGCCCTTCCGTCAATTCC, HPRT fwd CCTCCTCCTCTGCTCCGCCA and rev GGTTCATCATCACTAATCACGACGCCAG, CXCL10 fwd CCTGCATCAGCATTAGTAATCAACC and rev TGGATTCAGACATCTCTTCTCACC, IFIT1 fwd CCTTGCTGAAGTGTGGAGGA and rev CCTGCCTTAGGGGAAGCAAA, IFI27 fwd TGCTACAGTTGTGATTGGAGG and rev ACTGCAGAGTAGCCACAAGG, MX1 fwd CCAGCTCAGGGGCTTTGG and rev TTGGAATGGTGGCTGGATGG, STAT1 fwd AGTGTAAGTGAACACAGAAGAGTC and rev GTAACACGGGGATCTCAACAAG, RIG‐I fwd TGAAGCCATTGAAAGTTGGG and rev CCATCATCCCCTTAGTAGAGC, SIGLEC1 fwd ACCTGGAGGAAACTGACAGTGG and rev CTCAGTGTCACTGCCTGTCCTT, RSAD2 fwd CCAGTGCAACTACAAATGCGGC and rev CGGTCTTGAAGAAATGGCTCTCC, ISG15 fwd CTCTGAGCATCCTGGTGAGGAA and rev AAGGTCAGCCAGAACAGGTCGT, IFI44 fwd TCTATTCAATACTTCTCCTCTCAGATGATAG and rev TGAGCAAAGCCACATGTACCA. ISL1 fwd GCATGTTTGAAATGTGCGGA and rev TTTGATCCCGTACAACCTGA, MNX1 fwd CATGATCCTGCCTAAGATGCC and rev CGACAGGTACTTGTTGAGCTT, GFAP fwd CCTGCAGATTCGAGAAACCA and rev TGCCTCACATCACATCCTTG. Expression of mitochondrial genes MT‐ND1, MT‐CYB, MT‐COI, MT‐ATP6 and MT‐RNR1/12s was analyzed using previously published primer sequences [50].

The interferon signature score was calculated as demonstrated previously [31]. An IFN score above the 2.5× SD of the control group (> 12.49) was considered increased.

### 5.10 Neurofilament in Supernatant (ELLA)

NfL levels in iPSC supernatants were measured with the Ella microfluidic system using the Simple Plex Human NF‐L Cartridge (BioTechne, Minneapolis, USA) according to the manufacturer´s instructions. Quality control samples were included in all runs to monitor assay performance.

### 5.11 Post Mortem

Postmortem human tissue was obtained from ALS patients at the department of Neuropathology of the Amsterdam UMC (University of Amsterdam, the Netherlands). All patients with ALS fulfilled El Escorial criteria for diagnosis, as reviewed independently by two neuropathologists. ALS subjects died from respiratory failure or euthanasia. Tissues obtained from patients who died because of non‐neurological diseases were used as controls. No signs of infection before death were detected in both ALS and control patients included in the study. Informed consent was obtained for the use of brain tissue and access to medical records for research purposes; approval was obtained from the relevant local ethical committees for medical research. All autopsies were performed within 10 h after death. Paraffin‐embedded tissues were sectioned at 6 µm and mounted on pre‐coated glass slides (StarFrost, Waldemar Knittel Glasbearbeitungs GmbH, Braunschweig, Germany). Healthy control patients without prior neurological disease and two ALS patient with a *FUS‐*R521C mutation were analyzed. Spinal cord sections on the cervical, thoracic, and lumbar levels were processed as follows: At first, the sections were deparaffinized in xylene for 20 min and then rehydrated in 100%, 96%, and 70% ethanol for 5 min each followed by endogenous peroxidase quenching (0.3% H_2_O_2_ in methanol) for 20 min. Antigen retrieval was performed in these sections by heating them in citrate buffer, pH 6 (DAKO), for 20 min in a pressure cooker. After washing in PBS, sections were incubated with primary for 1 h at room temperature or 4°C overnight. After washing in PBS, sections were incubated with the appropriate secondary antibody (ImmunoLogic, Duiven, The Netherlands) for 30 min at room temperature. DAB reagent (ImmunoLogic, ready to use) was used to visualize antibody binding. The sections were then counter‐stained with 6% hematoxylin for 3 min. All procedures were performed at room temperature.

### 5.12 RNA‐Sequencing and Analysis

RNA‐seq analysis of somatodendritic (SD) and axonal compartments was performed as described in detail in Zimyanin V., Dash BP et al. [27]. The generation of human NPCs and sMNs was accomplished following a modified protocol from Reinhardt et al. [48] and is described in detail in Naumann&Pal, et al. [14]. Briefly, cells were grown in Xona (RD900) microfluidic devices (7–10 per sample per phenotype) and total RNA isolated from either SD or axonal compartment after 2 weeks of growth. Total RNA was sequenced (at Biotech Deep sequencing facility, TU, Dresden) on an Illumina HiSeq 2500 sequencing system using Illumina sequencing libraries. An average of about 30 million 75 base pairs long single‐end reads were produced for each sample for RNA‐seq. The sequencing reads were aligned to the human hg38/GRCh38 reference genome and EnsEMBL assembly v81 gene annotation model using GSNAP aligner (v2017‐11‐15)  [51] with splice‐junction support from annotated genes (EnsEMBL v81), respectively. A table of raw read counts per gene was obtained based on the overlap of the uniquely mapped reads with annotated human genes (EnsEMBL v81) using featureCounts (v1.5.2) [52]. Data normalization and differential expression were analyzed using the DESeq2 R package v1.16.1 [53] with a false discovery rate (FDR)/adjusted *p*‐value ≤ 0.05 and log_2_FC ≥ 1 or log_2_FC ≤ −1 as the significant cutoff. The PPI networks were performed with the Search Tool for the Retrieval of Interacting Genes (STRING) database v12.0 [54]. The datasets analyzed during the current study are available at GSE276214.

For further characterization of neuronal differentiation, we analyzed publicly available FASTQ files from the NCBI GEO database (GEO: GSE272827). This dataset contains transcriptome data of our isogenic pair that was analyzed in a previous study [55]. The cells were differentiated using the protocol described and published by our research group [14]. Raw sequencing data were obtained from the Sequence Read Archive (SRA) using the SRA Toolkit (v3.2.0). SRA files were downloaded using prefetch and converted to FASTQ format using fasterq‐dump. Sequencing data were processed using the nf‐core/rnaseq pipeline (v3.18.0) **([** 56 **])** as part of the Nextflow workflow (v24.10.5) [57].The pipeline performed quality control using FastQC, read trimming with Trim Galore (v0.6.10), and alignment to the human reference genome (GRCh38) using STAR (v2.7.11b) [58]. Gene‐level read counts were quantified using featureCounts from the Subread package (v2.0.1) [52].

Raw gene‐level counts were normalized using the DESeq2 package (v1.46.0) [53] in R (v4.2.2). Size factors were calculated to account for differences in library size and sequencing depth across samples using the “poscounts” method. We accounted for batch effects and phenotype (FUS‐WT_eGFP vs. FUS‐p.P525L_eGFP) in the design formula (∼ batch + phenotype). Dispersion estimates were calculated to model the mean‐variance relationship across genes. The normalized data were used for downstream visualization and clustering analysis.

### 5.13 Cell Type Analysis of GSE272827, GSE168831 and GSE158264

Cell type marker gene expression was visualized using combined heatmaps generated in Python (v3.13). We compared two independent datasets: RNA‐seq data from our iPSC‐derived FUS‐WT_eGFP/FUS‐p.P525L_eGFP motor neurons (GSE272827) and RNA‐seq data from iPSC‐derived CTRL/FUS‐p.R521C/FUS‐p.R521L motor neurons (GSE168831), and microarray data from iPSC‐derived CTRL/FUS‐p.R521C/FUS‐p.R521L motor neurons (GSE158264). For RNA‐seq data, DESeq2‐normalized counts were log2‐transformed using the formula log2(count + 1) to stabilize variance. Microarray data were RMA (Robust Multi‐array Average) normalized and already log2‐transformed, and were used directly for visualization. We selected a panel of cell type‐specific markers based on published literature, including three astroglia markers (AQP4, GFAP, S100B) [59, 60], 19 motor neuron markers including both postmitotic motor neuron‐specific genes (*LHX3, FOXP1, ETV4, LMO4, ALCAM, SMAD1, ZEB2*) and pan‐neuronal markers (*SYT1, MAP2, STMN2, NEFM, SNAP25, SYT4, NCAM1, GAP43, L1CAM, TUBB3, NEFL, ELAVL3*) [61], three microglia markers (*PTPRC, CX3CR1, TMEM119*), and three oligodendrocyte markers (*MOG, OLIG2, SOX10*). Each sample replicate is displayed individually in the heatmap without averaging.

Heatmaps were generated using Matplotlib (v3.10.11) (15), NumPy (v2.1.3) (17), and Pandas (v2.2.3) (18). The combined heatmap displays both datasets side‐by‐side, with RNA‐seq samples in the upper section and microarray samples in the lower section, separated by a thick horizontal line for visual distinction. Expression values were displayed using a blue‐white‐red color scheme with a fixed scale (0–16) to enable direct comparison between datasets, where blue indicates low expression, white indicates intermediate expression, and red indicates high expression. All individual sample replicates are displayed separately without averaging, allowing visualization of biological variability within each genotype group. Genes were grouped by cell type (astroglia, motor neuron, microglia, and oligodendrocyte) and displayed in columns. Dataset labels (GSE272827 RNA‐seq, GSE168831 RNA‐seq, and GSE158264 Microarray) were positioned vertically on the left side, while cell type labels were positioned horizontally above the gene columns. Thick horizontal separators were added to distinguish between different genotype groups within each dataset. Grey values indicate genes that showed undetectable expression in the count matrix.

## Author Contributions

Conceptualization (M.N., A.H.); Data curation (M.N., T.W., S.K., V.Z.); Formal Analysis (M.N., T.W., A.S., A.K., N.D.S., A.He., M.K., S.K., V.Z., P.O., B.P.D.); Funding acquisition (A.H.); Methodology (M.N., D.G., V.Z., M.K., S.K., M.L.K., A.He., A.C., B.J.W., P.O.); Project administration (A.H.); Resources (M.N., E.A., K.P., R.G., V.Z., M.K., H.G., F.P., M.L.K., S.K., D.B., S.P., T.B., J.S., A.He., A.R., T.G., P.Ö., B.J.W., A.H.); Software (M.N., H.G., D.G., V.Z., B.P.D.); Supervision (A.H.); Validation (M.N., T.W., D.G.); Visualization (M.N., A.H.); Writing – original draft (M.N., A.H.); Writing – review & editing (all authors)

## Funding

A.H. is supported by the Hermann und Lilly Schilling‐Stiftung für medizinische Forschung im Stifterverband. M.N. is supported by the Clinician Scientist program of the Medical Faculty of the University of Rostock (RAS). DFG Großgeräteantrag für LSM 800 Airyscan. FKZ: INST 264/175‐1 FUGG. E.A. is supported by ALS Stichting (grant “ALS Tissue Bank – NL”). J.S. was supported by the Technische Universität Dresden. Part of the work (author B.P.D) was funded by the framework of the Professorinnenprogramm III (University of Rostock) of the German federal and state governments. M.L.‐K. is supported by German Research Foundation (DFG) grants CRC237 369799452/B21, CRC237 369799452/A11, CRC369 501752319/C06 and by grants of the German Federal Ministry of Education and Research (BMBF) 01GM2206C (GAIN) and 01GL2405H (DZKJ).

## Institutional Review Board Statement

The performed procedures were in accordance with the Declaration of Helsinki (WMA, 1964) and approved by the Ethical Committee of the Technische Universität Dresden, Germany (EK 393122012 and EK 45022009) and Rostock (A2019‐0137).

## Consent

Written informed consent was obtained from all participants including for publication of any research results.

## Conflicts of Interest

A. H. has received personal fees and non‐financial support from Biogen, IFT Pharma, Zambon and Desitin during the conduct of the study outside of the submitted work. R. G. has received honoraria from Biogen as an advisory board member and for lectures and as a consultant and advisory board member from Hoffmann‐La Roche. He also received travel expenses and research support from Biogen. S.P. has received personal fees and non‐financial support from Amylyx, Biogen, Ferrer, Italfarmaco, and Zambon during the conduct of the study outside of the submitted work. MN has received travel expenses from Italfarmaco during the conduct of the study outside of the submitted work. PO received research support from the Cure Alzheimer Fund, ALS Association (24‐SGP‐691, 23‐PPG‐674‐2), ALS Finding a Cure, the Charcot Foundation, the DZNE Innovation‐to‐Application program and consulting fees from LifeArc and Fundamental Pharma. EA has no conflicts of interest.

## Supporting information




**Supporting File**: advs73807‐sup‐0001‐SuppMat.docx.

## Data Availability

We have included all relevant data in the manuscript. The RNA‐seq datasets were obtained from GEO database (Accession No. GSE276214) (Zimyanin V., Dash BP). More details are available from the authors.

## References

[1] J. H. Weishaupt , T. Hyman , and I. Dikic , “Common Molecular Pathways in Amyotrophic Lateral Sclerosis and Frontotemporal Dementia,” Trends in Molecular Medicine 22, no. 9 (2016): 769–783, 10.1016/j.molmed.2016.07.005. 27498188

[2] M. F. Gulen , N. Samson , A. Keller , et al., “cGAS–STING Drives Ageing‐Related Inflammation and Neurodegeneration,” Nature 620, no. 7973 (2023): 374–380, 10.1038/s41586-023-06373-1. 37532932 PMC10412454

[3] X. Xie , G. Ma , X. Li , et al., “Activation of Innate Immune cGAS‐STING Pathway Contributes to Alzheimer'sPathogenesis in 5×FAD Mice,” Nature Aging 3 (2023): 202–212, 10.1038/s43587-022-00337-2. 37118112

[4] C. H. Yu , S. Davidson , C. R. Harapas , et al., “TDP‐43 Triggers Mitochondrial DNA Release via mPTP to Activate cGAS/STING in ALS,” Cell 183, no. 3 (2020): 636–649.e18, 10.1016/j.cell.2020.09.020. 33031745 PMC7599077

[5] A. Ablasser , M. Goldeck , T. Cavlar , et al., “cGAS Produces a 2′‐5′‐Linked Cyclic Dinucleotide Second Messenger That Activates STING,” Nature 498, no. 7454 (2013): 380–384, 10.1038/nature12306. 23722158 PMC4143541

[6] B. Wu and S. Hur , “How RIG‐I Like Receptors Activate MAVS,” Current Opinion in Virology 12 (2015): 91–98, 10.1016/j.coviro.2015.04.004. 25942693 PMC4470786

[7] S. Liu , X. Cai , J. Wu , et al., “Phosphorylation of Innate Immune Adaptor Proteins MAVS, STING, and TRIF Induces IRF3 Activation,” Science 347, no. 6227 (2015): aaa2630, 10.1126/science.aaa2630. 25636800

[8] M. Al Hamrashdi and G. Brady , “Regulation of IRF3 Activation in Human Antiviral Signaling Pathways,” Biochemical Pharmacology 200 (2022): 115026, 10.1016/j.bcp.2022.115026. 35367198

[9] W. Pang and F. Hu , “C9ORF72 Suppresses JAK‐STAT Mediated Inflammation,” Iscience 26 (2023): 106579, 10.1016/j.isci.2023.106579. 37250330 PMC10214391

[10] S. Rodriguez , A. Sahin , B. R. Schrank , et al., “Genome‐Encoded Cytoplasmic Double‐Stranded RNAs, Found in C9ORF72 ALS‐FTD Brain, Propagate Neuronal Loss,” Science Translational Medicine 13, no. 601 (2021), 10.1126/scitranslmed.aaz4699. PMC8779652 34233951

[11] M. E. McCauley , J. G. O'Rourke , A. Yanez , et al., “C9orf72 in Myeloid Cells Suppresses STING‐Induced Inflammation,” Nature 585, no. 7823 (2020): 96–101, 10.1038/s41586-020-2625-x. 32814898 PMC7484469

[12] H. Y. Tan , Y. K. Yong , Y. C. Xue , et al., “cGAS and DDX41‐STING Mediated Intrinsic Immunity Spreads Intercellularly to Promote Neuroinflammation in SOD1 ALS Model,” Iscience 25, no. 6 (2022): 104404, 10.1016/j.isci.2022.104404. 35712074 PMC9194172

[13] C. Marques , A. Held , K. Dorfman , et al., “Neuronal STING Activation in Amyotrophic Lateral Sclerosis and Frontotemporal Dementia,” Acta Neuropathologica 147, no. 1 (2024): 56, 10.1007/s00401-024-02688-z. 38478117 PMC10937762

[14] M. Naumann , A. Pal , A. Goswami , et al., “Impaired DNA Damage Response Signaling by FUS‐NLS Mutations Leads to Neurodegeneration and FUS Aggregate Formation,” Nature Communications 9, no. 1 (2018): 335, 10.1038/s41467-017-02299-1. PMC5780468 29362359

[15] V. L. Zimyanin , A. M. Pielka , H. Glass , et al., “Live Cell Imaging of ATP Levels Reveals Metabolic Compartmentalization Within Motoneurons and Early Metabolic Changes in FUS ALS Motoneurons,” Cells 12, no. 10 (2023): 1352, 10.3390/cells12101352. 37408187 PMC10216752

[16] W. Guo , M. Naujock , L. Fumagalli , et al., “HDAC6 Inhibition Reverses Axonal Transport Defects in Motor Neurons Derived From FUS‐ALS Patients,” Nature communications 8, no. 1 (2017): 861, 10.1038/s41467-017-00911-y. PMC5636840 29021520

[17] W. Guo , H. Wang , A. Kumar Tharkeshwar , et al., “CRISPR/Cas9 Screen in Human iPSC‐Derived Cortical Neurons Identifies NEK6 as a Novel Disease Modifier of C9orf72 Poly(PR) Toxicity,” Alzheimer's & Dementia 19, no. 4 (2023): 1245–1259, 10.1002/alz.12760. PMC9943798 35993441

[18] K. De Vos , F. Severin , F. Van Herreweghe , et al., “Tumor Necrosis Factor Induces Hyperphosphorylation of Kinesin Light Chain and Inhibits Kinesin‐Mediated Transport of Mitochondria,” The Journal of Cell Biology 149, no. 6 (2000): 1207–1214, 10.1083/jcb.149.6.1207. 10851018 PMC2175118

[19] B. Viengkhou and M. J. Hofer , “Breaking Down the Cellular Responses to Type I Interferon Neurotoxicity in the Brain,” Frontiers in Immunology 14 (2023): 1110593, 10.3389/fimmu.2023.1110593. 36817430 PMC9936317

[20] A. Held , M. Adler , C. Marques , et al., “iPSC Motor Neurons, but Not Other Derived Cell Types, Capture Gene Expression Changes in Postmortem Sporadic ALS Motor Neurons,” Cell Reports 42, no. 9 (2023): 113046, 10.1016/j.celrep.2023.113046. 37651231 PMC10622181

[21] S. L. Rulten , A. Rotheray , R. L. Green , et al., “PARP‐1 Dependent Recruitment of the Amyotrophic Lateral Sclerosis‐Associated Protein FUS/TLS to Sites of Oxidative DNA Damage,” Nucleic Acids Research 42, no. 1 (2014): 307–314, 10.1093/nar/gkt835. 24049082 PMC3874156

[22] S. M. Haag , M. F. Gulen , L. Reymond , et al., “Targeting STING With Covalent Small‐Molecule Inhibitors,” Nature 559, no. 7713 (2018): 269–273, 10.1038/s41586-018-0287-8. 29973723

[23] A. Dhir , S. Dhir , L. S. Borowski , et al., “Mitochondrial Double‐Stranded RNA Triggers Antiviral Signalling in Humans,” Nature 560, no. 7717 (2018): 238–242, 10.1038/s41586-018-0363-0. 30046113 PMC6570621

[24] Y. Wang , S. Yuan , X. Jia , et al., “Mitochondria‐Localised ZNFX1 functions as a dsRNA Sensor to Initiate Antiviral Responses Through MAVS,” Nature Cell Biology 21, no. 11 (2019): 1346–1356, 10.1038/s41556-019-0416-0. 31685995

[25] K. E. Irwin , U. Sheth , P. C. Wong , and T. F. Gendron , “Fluid Biomarkers for Amyotrophic Lateral Sclerosis: A Review,” Molecular Neurodegeneration 19, no. 1 (2024): 9, 10.1186/s13024-023-00685-6. 38267984 PMC10809579

[26] A. Ostojic , R. Vrhovac , and S. Verstovsek , “Ruxolitinib: A New JAK1/2 Inhibitor That Offers Promising Options for Treatment of Myelofibrosis,” Future Oncology 7, no. 9 (2011): 1035–1043, 10.2217/fon.11.81. 21919691 PMC5147419

[27] V. Zimyanin , B. P. Dash , D. Großmann , et al., “Axonal Transcriptome Reveals Upregulation of PLK1 as a Protective Mechanism in Response to Increased DNA Damage in FUS^P525L^ Spinal Motor Neurons,” BioRxiv (2024): 624439, 10.1101/2024.11.20.624439.

[28] N. A. Bonekamp , B. Peter , H. S. Hillen , et al., “Small‐Molecule Inhibitors of Human Mitochondrial DNA Transcription,” Nature 588, no. 7839 (2020): 712–716, 10.1038/s41586-020-03048-z. 33328633

[29] E. Arnaiz , A. Miar , A. G. Dias Junior , et al., “Hypoxia Regulates Endogenous Double‐Stranded RNA Production via Reduced Mitochondrial DNA Transcription,” Frontiers in Oncology 11 (2021): 779739, 10.3389/fonc.2021.779739. 34900733 PMC8651540

[30] E. C. Baechler , F. M. Batliwalla , G. Karypis , et al., “Interferon‐Inducible Gene Expression Signature in Peripheral Blood Cells of Patients With Severe Lupus,” Proceedings of the National Academy of Sciences 100, no. 5 (2003): 2610–2615, 10.1073/pnas.0337679100. PMC151388 12604793

[31] C. Wolf , N. Bruck , S. Koss , et al., “Janus Kinase Inhibition in Complement Component 1 Deficiency,” Journal of Allergy and Clinical Immunology 146, no. 6 (2020): 1439–1442.e5, 10.1016/j.jaci.2020.04.002. 32325142

[32] W. Dunker , X. Ye , Y. Zhao , L. Liu , A. Richardson , and J. Karijolich , “TDP‐43 Prevents Endogenous RNAs From Triggering a Lethal RIG‐I‐Dependent Interferon Response,” Cell Reports 35, no. 2 (2021): 108976, 10.1016/j.celrep.2021.108976. 33852834 PMC8109599

[33] M. Paget , C. Cadena , S. Ahmad , et al., “Stress Granules are Shock Absorbers That Prevent Excessive Innate Immune Responses to dsRNA,” Molecular Cell 83, no. 7 (2023): 1180–1196.e8, 10.1016/j.molcel.2023.03.010. 37028415 PMC10170497

[34] S. Maharana , S. Kretschmer , S. Hunger , et al., “SAMHD1 Controls Innate Immunity by Regulating Condensation of Immunogenic Self RNA,” Molecular Cell 82, no. 19 (2022): 3712–3728.e10, 10.1016/j.molcel.2022.08.031. 36150385

[35] S. S. Kim , L. Sze , C. Liu , and K. P. Lam , “The Stress Granule Protein G3BP1 Binds Viral dsRNA and RIG‐I to Enhance Interferon‐β Response,” Journal of Biological Chemistry 294, no. 16 (2019): 6430–6438, 10.1074/jbc.RA118.005868. 30804210 PMC6484135

[36] P. Manivannan , M. A. Siddiqui , and K. Malathi , “RNase L Amplifies Interferon Signaling by Inducing Protein Kinase R‐Mediated Antiviral Stress Granules,” Journal of Virology 94, no. 13 (2020), 10.1128/JVI.00205-20. PMC7307175 32295917

[37] B. Szewczyk , R. Gunther , J. Japtok , et al., “FUS ALS Neurons Activate Major Stress Pathways and Reduce Translation as an Early Protective Mechanism Against Neurodegeneration,” Cell Reports 42, no. 2 (2023): 112025, 10.1016/j.celrep.2023.112025. 36696267

[38] M. Kodavati , H. Wang , W. Guo , et al., “FUS Unveiled in Mitochondrial DNA Repair and Targeted Ligase‐1 Expression Rescues Repair‐Defects in FUS‐Linked Motor Neuron Disease,” Nature Communications 15, no. 1 (2024): 2156, 10.1038/s41467-024-45978-6. PMC10925063 38461154

[39] J. Deng , M. Yang , Y. Chen , et al., “FUS Interacts With HSP60 to Promote Mitochondrial Damage,” PLOS Genetics 11, no. 9 (2015): 1005357, 10.1371/journal.pgen.1005357. PMC4559378 26335776

[40] L. MacNair , S. Xiao , D. Miletic , et al., “MTHFSD and DDX58 are Novel RNA‐Binding Proteins Abnormally Regulated in Amyotrophic Lateral Sclerosis,” Brain 139 (2016): 86–100, 10.1093/brain/awv308. 26525917

[41] H. Honda , K. Yagita , H. Arahata , et al., “Increased Expression of Human Antiviral Protein MxA in FUS Proteinopathy In Amyotrophic Lateral Sclerosis,” Brain Pathology 34, no. 2 (2024): 13191, 10.1111/bpa.13191. PMC10901610 37586842

[42] F. Ma , B. Li , Y. Yu , S. S. Iyer , M. Sun , and G. Cheng , “Positive Feedback Regulation of Type I Interferon by the Interferon‐Stimulated Gene STING,” EMBO Reports 16, no. 2 (2015): 202–212, 10.15252/embr.201439366. 25572843 PMC4328747

[43] H. Wang , W. Guo , J. Mitra , et al., “Mutant FUS Causes DNA Ligation Defects to Inhibit Oxidative Damage Repair in Amyotrophic Lateral Sclerosis,” Nature Communications 9, no. 1 (2018): 3683, 10.1038/s41467-018-06111-6. PMC6134028 30206235

[44] Q. Deng , C. J. Holler , G. Taylor , et al., “FUS is Phosphorylated by DNA‐PK and Accumulates in the Cytoplasm After DNA Damage,” Journal of Neuroscience 34, no. 23 (2014): 7802–7813, 10.1523/JNEUROSCI.0172-14.2014. 24899704 PMC4044245

[45] P. K. Gupta , S. Prabhakar , S. Sharma , and A. Anand , “Vascular Endothelial Growth Factor‐A (VEGF‐A) and Chemokine Ligand‐2 (CCL2) in Amyotrophic Lateral Sclerosis (ALS) Patients,” Journal of Neuroinflammation 8 (2011): 47, 10.1186/1742-2094-8-47. 21569455 PMC3117705

[46] K. E. Irwin , P. Jasin , K. E. Braunstein , et al., “A Fluid Biomarker Reveals Loss of TDP‐43 Splicing Repression in Presymptomatic ALS–FTD,” Nature Medicine 30, no. 2 (2024): 382–393, 10.1038/s41591-023-02788-5. PMC10878965 38278991

[47] M. Chatterjee , S. Ozdemir , C. Fritz , et al., “Plasma Extracellular Vesicle Tau and TDP‐43 as Diagnostic Biomarkers in FTD and ALS,” Nature Medicine 30, no. 6 (2024): 1771–1783, 10.1038/s41591-024-02937-4. PMC11186765 38890531

[48] P. Reinhardt , M. Glatza , K. Hemmer , et al., “Derivation and Expansion Using Only Small Molecules of Human Neural Progenitors for Neurodegenerative Disease Modeling,” PLoS ONE 8, no. 3 (2013): 59252, 10.1371/journal.pone.0059252. PMC3606479 23533608

[49] C. B. Pantazis , A. Yang , E. Lara , et al., “A Reference Human Induced Pluripotent Stem Cell Line for Large‐Scale Collaborative Studies,” Cell Stem Cell 29, no. 12 (2022): 1685–1702.e22, 10.1016/j.stem.2022.11.004. 36459969 PMC9782786

[50] L. Wallace , S. Mehrabi , M. Bacanamwo , X. Yao , and F. O. Aikhionbare , “Expression of Mitochondrial Genes MT‐ND1, MT‐ND6, MT‐CYB, MT‐COI, MT‐ATP6, and 12S/MT‐RNR1 in Colorectal Adenopolyps,” Tumor Biology 37, no. 9 (2016): 12465–12475, 10.1007/s13277-016-5101-3. 27333991 PMC5661973

[51] T. D. Wu and S. Nacu , “Fast and SNP‐Tolerant Detection of Complex Variants and Splicing in Short Reads,” Bioinformatics 26, no. 7 (2010): 873–881, 10.1093/bioinformatics/btq057. 20147302 PMC2844994

[52] Y. Liao , G. K. Smyth , and W. Shi , “FeatureCounts: An Efficient General Purpose Program for Assigning Sequence Reads to Genomic Features,” Bioinformatics 30, no. 7 (2014): 923–930, 10.1093/bioinformatics/btt656. 24227677

[53] M. I. Love , W. Huber , and S. Anders , “Moderated Estimation of Fold Change and Dispersion for RNA‐Seq Data With DESeq2,” Genome Biology 15, no. 12 (2014): 550, 10.1186/s13059-014-0550-8. 25516281 PMC4302049

[54] D. Szklarczyk , A. Franceschini , S. Wyder , et al., “STRING v10: Protein–Protein Interaction Networks, Integrated Over the Tree of Life,” Nucleic Acids Research 43 (2015): D447–D452, 10.1093/nar/gku1003. 25352553 PMC4383874

[55] B. Szewczyk , V. Zimyanin , J. Japtok , et al., “Activation of Polo‐Like Kinase 1 Correlates With Selective Motor Neuron Vulnerability in Familial ALS,” Cell Reports 44, no. 9 (2025): 116113, 10.1016/j.celrep.2025.116113. 40857153

[56] P. A. Ewels , A. Peltzer , S. Fillinger , et al., “The Nf‐Core Framework for Community‐Curated Bioinformatics Pipelines,” Nature Biotechnology 38, no. 3 (2020): 276–278, 10.1038/s41587-020-0439-x. 32055031

[57] P. Di Tommaso , M. Chatzou , E. W. Floden , P. P. Barja , E. Palumbo , and C. Notredame , “Nextflow Enables Reproducible Computational Workflows,” Nature Biotechnology 35, no. 4 (2017): 316–319, 10.1038/nbt.3820. 28398311

[58] A. Dobin , C. A. Davis , F. Schlesinger , et al., “STAR: Ultrafast Universal RNA‐Seq Aligner,” Bioinformatics 29, no. 1 (2013): 15–21, 10.1093/bioinformatics/bts635. 23104886 PMC3530905

[59] M. Y. Batiuk , A. Martirosyan , J. Wahis , et al., “Identification of Region‐Specific Astrocyte Subtypes at Single Cell Resolution,” Nature Communications 11, no. 1 (2020): 1220, 10.1038/s41467-019-14198-8. PMC7058027 32139688

[60] Z. Zhang , Z. Ma , W. Zou , et al., “The Appropriate Marker for Astrocytes: Comparing the Distribution and Expression of Three Astrocytic Markers in Different Mouse Cerebral Regions,” BioMed Research International 2019 (2019): 9605265, 10.1155/2019/9605265. 31341912 PMC6613026

[61] O. J. Ziff , J. Neeves , J. Mitchell , et al., “Integrated Transcriptome Landscape of ALS Identifies Genome Instability Linked to TDP‐43 Pathology,” Nature Communications 14, no. 1 (2023): 2176, 10.1038/s41467-023-37630-6. PMC10119258 37080969

