# Supplementary material for: RIG‐I Mediated Neuron‐Specific IFN Type 1 Signaling in FUS‐ALS Induces Neurodegeneration and Offers New Biomarker‐Driven Individualized Treatment Options for (FUS‐)ALS
Source: Adv Sci (Weinh). 2026 Jan 28;13(18):e17135. doi: 10.1002/advs.202417135 (PMC13042818; doi:10.1002/advs.202417135)
Supplement: Supplementary file 1 — Supporting File: advs73807‐sup‐0001‐SuppMat.docx. [file ADVS-13-e17135-s001.docx]

**Supplemental Material**

**Title: “RIG-I mediated neuron-specific IFN type 1 signaling in FUS-ALS induces neurodegeneration and offers new biomarker-driven individualized treatment options for (FUS-)ALS.”**

Marcel Naumann (1), Theresa M. Wierschin (1), Stefanie Kretschmer (2), Banaja P. Dash (1), Aaron Held(3), Andrea Salzinger (3), Kevin Peikert (1,4), Anže Karlek( 1), Hannes Glaß (1), Dajana Großmann (1), René Günther(5,6), Susanne Petri ( 7), Annekathrin Rödiger (8), David Brenner (9), Francisco Pan-Montojo (10,11,12), Eleonora Aronica (13), Markus Kipp( 4,14), Vitaly Zimyanin (15,16), Jared Sterneckert (17), Torsten Grehl (18), Noah D. Seebacher (19), Tobias M. Böckers (19,20), Alberto Catanese ( 19, 20,21), Brian J. Wainger (3, 22, 23), Patrick Oeckl (9,20), Min Ae Lee-Kirsch( 2,24,25), Andreas Hermann (1,4, 26)

1 Translational Neurodegeneration Section “Albrecht Kossel”, Department of Neurology, University Medical Center Rostock, University of Rostock, Rostock, Germany.

2 Department of Pediatrics, Medizinische Fakultät Carl Gustav Carus, Technische Universität Dresden, Dresden, Germany.

3 Department of Neurology, Sean M. Healey & AMG Center for ALS, Massachusetts General Hospital, Harvard Medical School, Boston, MA , USA

4 Center for Transdisciplinary Neurosciences Rostock (CTNR), University Medical Center Rostock, University of Rostock, Rostock, German

5 Department of Neurology, University Hospital Carl Gustav Carus at Technische Universität Dresden, Dresden, Germany.

6 German Center for Neurodegenerative DIseases (DZNE) Dresden, Dresden, Germany.

7 Department of Neurology, Hannover Medical School, Hannover, Germany.

8 Department of Neurology, Jena University Hospital, Jena, Germany.

9 University Hospital Ulm, Department of Neurology, Ulm, Germany.

10 Dept. of Psychiatry and Psychotherapy at the Klinikum LMU, Munich, Germany and Neurologische Klinik Sorpesee, Sundern, Germany.

11 Neurosciences Area, Biogipuzkoa Health Research Institute, 20014 Donostia/San Sebastian, Spain

12 CIBERNED, ISCIII (CIBER, Carlos III Institute, Spanish Ministry of Sciences and Innovation), 28031, Madrid, Spain.

13 Amsterdam UMC, University of Amsterdam, Department of (Neuro)Pathology, Amsterdam Neuroscience, Amsterdam, The Netherlands.

14 Rostock University Medical Center, Institute of Anatomy, Rostock, Germany.

15 Department of Molecular Physiology and Biological Physics, School of Medicine, University of Virginia, Charlottesville, VA, 22903, USA;

16 Center for Membrane and Cell Physiology, School of Medicine, University of Virginia, Charlottesville, VA, 22903, USA

17 Center for Regenerative Therapies TU Dresden (CRTD) and the Medizinische Fakultät Carl Gustav Carus, Technische Universität Dresden, Dresden, Germany.

18 Department of Neurology, Alfred Krupp Hospital, Essen, Germany.

19 Institute of Anatomy and Cell Biology, University of Ulm, Ulm, Germany.

20 German Center for Neurodegenerative Diseases (DZNE) Ulm, Ulm, Germany.

21 Institute of Neuroanatomy, University Clinic Aachen, 52074 Aachen, Germany

22 Department of Anesthesiology, Critical Care and Pain Medicine, Massachusetts General Hospital, Boston MA 02114, USA

23 Broad Institute of Harvard University and MIT, Cambridge MA 02142, USA

24 University Centre for Rare Diseases, University Hospital Carl Gustav Carus, Technische Universität Dresden, Dresden, Germany

25 German Center for Child and Adolescent Health (DZKJ), partner site Leipzig/Dresden, Dresden, Germany.

26 German Center for Neurodegenerative Diseases (DZNE) Rostock/Greifswald, Rostock, Germany.


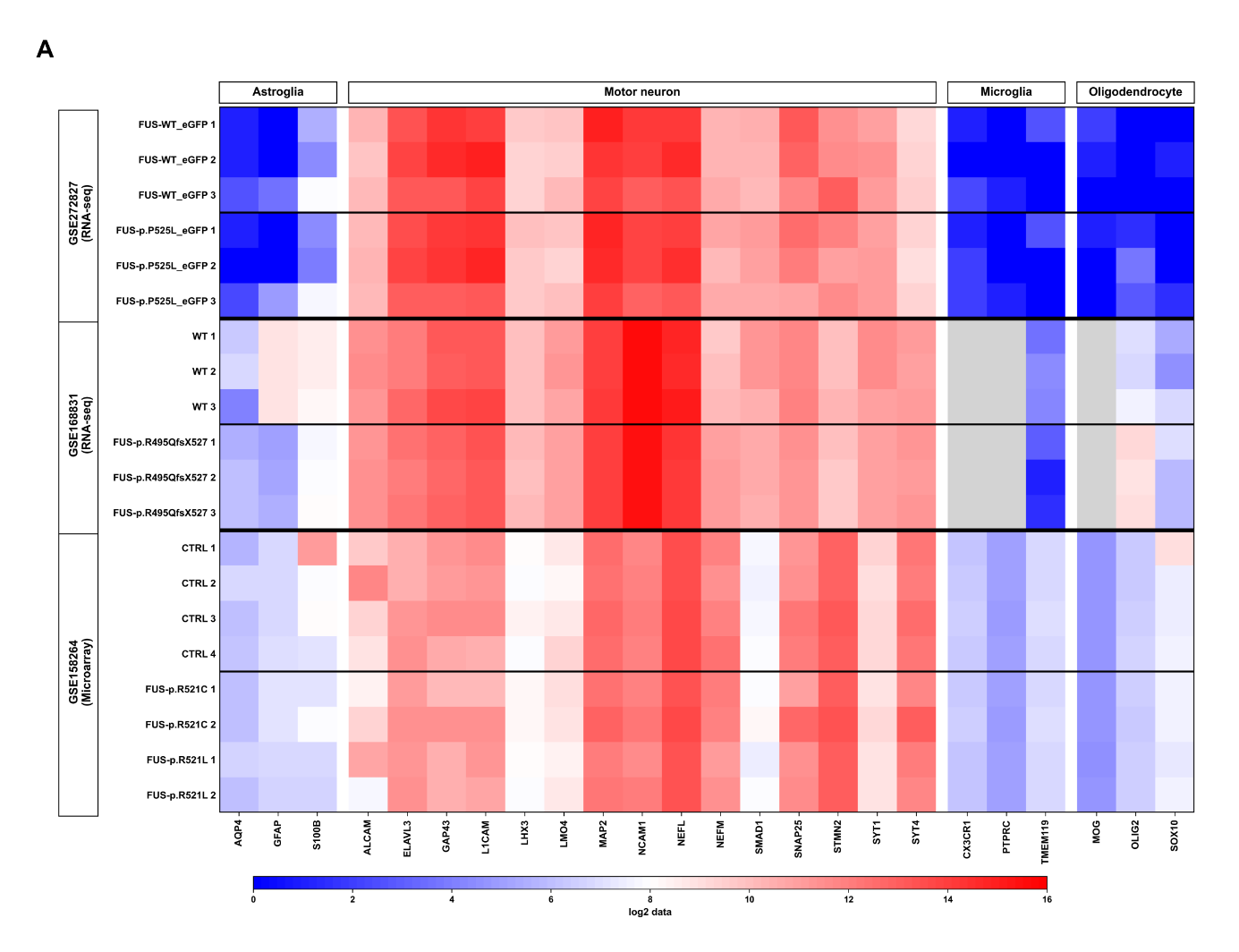


**Supplement Figure 1: Cell type marker gene expression across RNA-seq, microarray datasets and IF of neuronal markers.** (A) Combined heatmap displaying expression of astroglia, motor neuron, microglia, and oligodendrocyte marker genes in three independent datasets. Upper section: Individual replicates of FUS-WT_eGFP and FUS-p.P525L_eGFP samples from RNA-seq data (GSE272827), with three replicates per genotype displayed separately. Middle section: Individual replicates of WT and FUS-I samples with FUS-p.R495QfsX527 frameshift mutation from RNA-seq data (GSE168831). Lower section: Individual replicates of CTRL, FUS-p.R521C, and FUS-p.R521L samples from microarray data (GSE158264). Expression values are displayed on a fixed log2 scale (0-16) to enable direct comparison between datasets. RNA-seq values represent log2-transformed normalized counts (log2(count + 1)); microarray values represent RMA-normalized log2-transformed intensities. Each row represents a single biological replicate without averaging. Columns show marker genes grouped by cell type: astroglia (*AQP4, GFAP, S100B*), motor neuron (*GAP43, L1CAM, LHX3, LMO4, MAP2, NCAM1, NEFM, SMAD1, SNAP25, STMN2, SYT1, SYT4, ETV4, FOXP1, ALCAM, ZEB2, NEFL, ELAVL3*), microglia (*CX3CR1, PTPRC, TMEM119*), and oligodendrocyte (*MOG, OLIG2, SOX10*). Blue indicates low expression; red indicates high expression. Grey values indicate genes with undetectable expression in count matrix. Thick horizontal lines separate the three datasets and different genotype groups within each dataset.


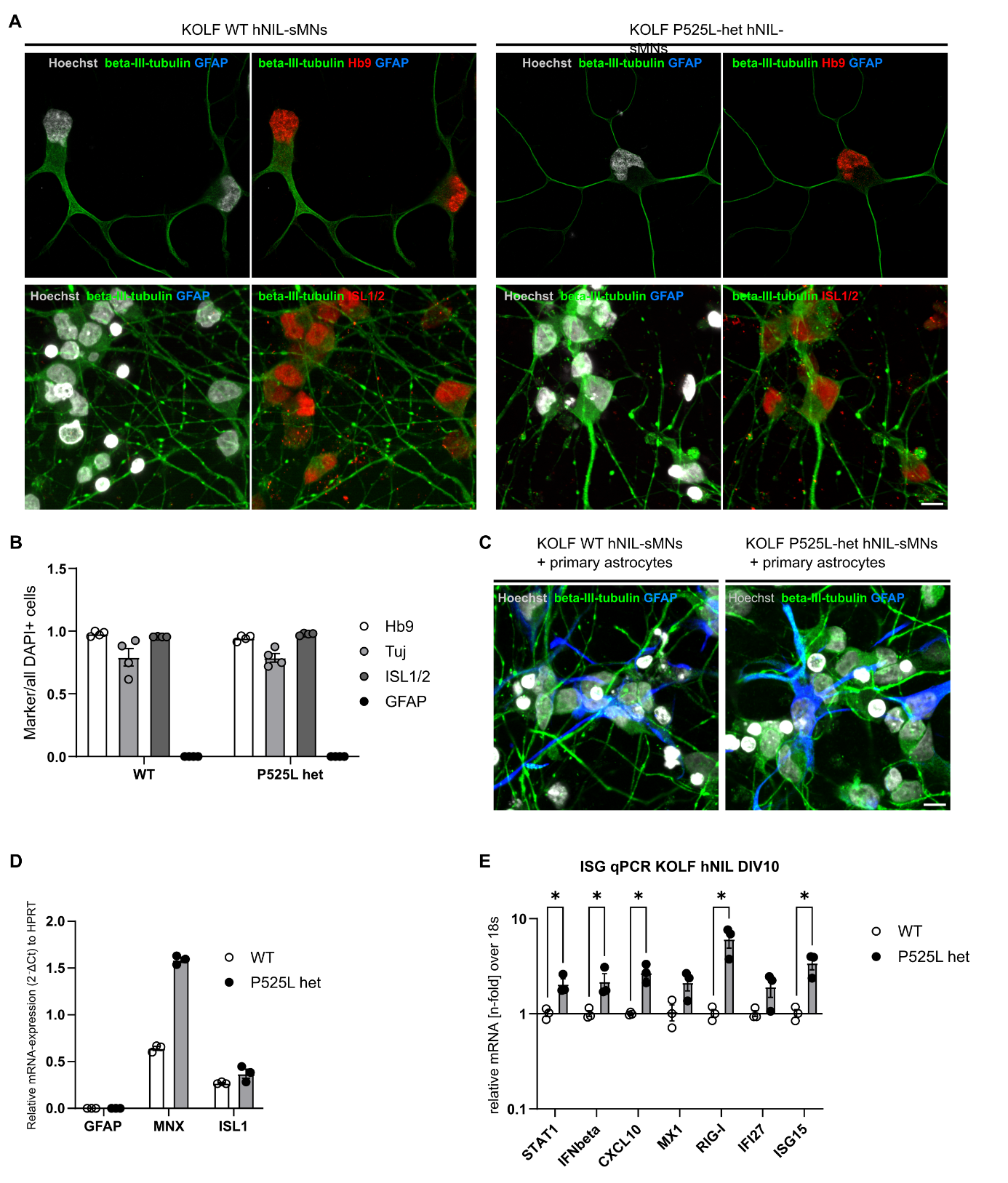


**Supplement Figure 2: Characterisation and analysis of innate immune signalling in KOLF P525L-het hNIL-sMNs at day 10 after differentiation induction.** (A) Conventional confocal LSM IF panel for markers of motoneuron development and nuclear staining with Hoechst. The vast majority of cells (nuclei) were either positive for the early motoneuron markers Hb9 and ISL1/2, while most of them also stained for the general neuronal marker beta-III-tubulin. Of note, no GFAP signal was detected. Scale bar = 10µM. (B) Quantification of (A), n= 4 biological replicates. Both cell lines (KOLF WT and KOLF FUS-P525L) showed a robust high motoneuron yield only 10 days after hNIL induction in iPSCs with doxycycline. (C) Positive control for GFAP signal in the culture. ScienCell primary astrocytes were added to the culture on Day 6 of hNIL-induction after removal of aphidicolin. (D) qPCR for mRNA of GFAP and sMN markers MNX and ISL1 is demonstrated as relative expression to the reference gene HPRT (2^(-delta-CT)). While MNX and ISL1 are robustly expressed in both cell lines, there was no expression of GFAP. (E) Significant upregulation of indicated ISGs in FUS mutant sMN compared to the isogenic KOLF WT, unpaired t-test, n=3).

**
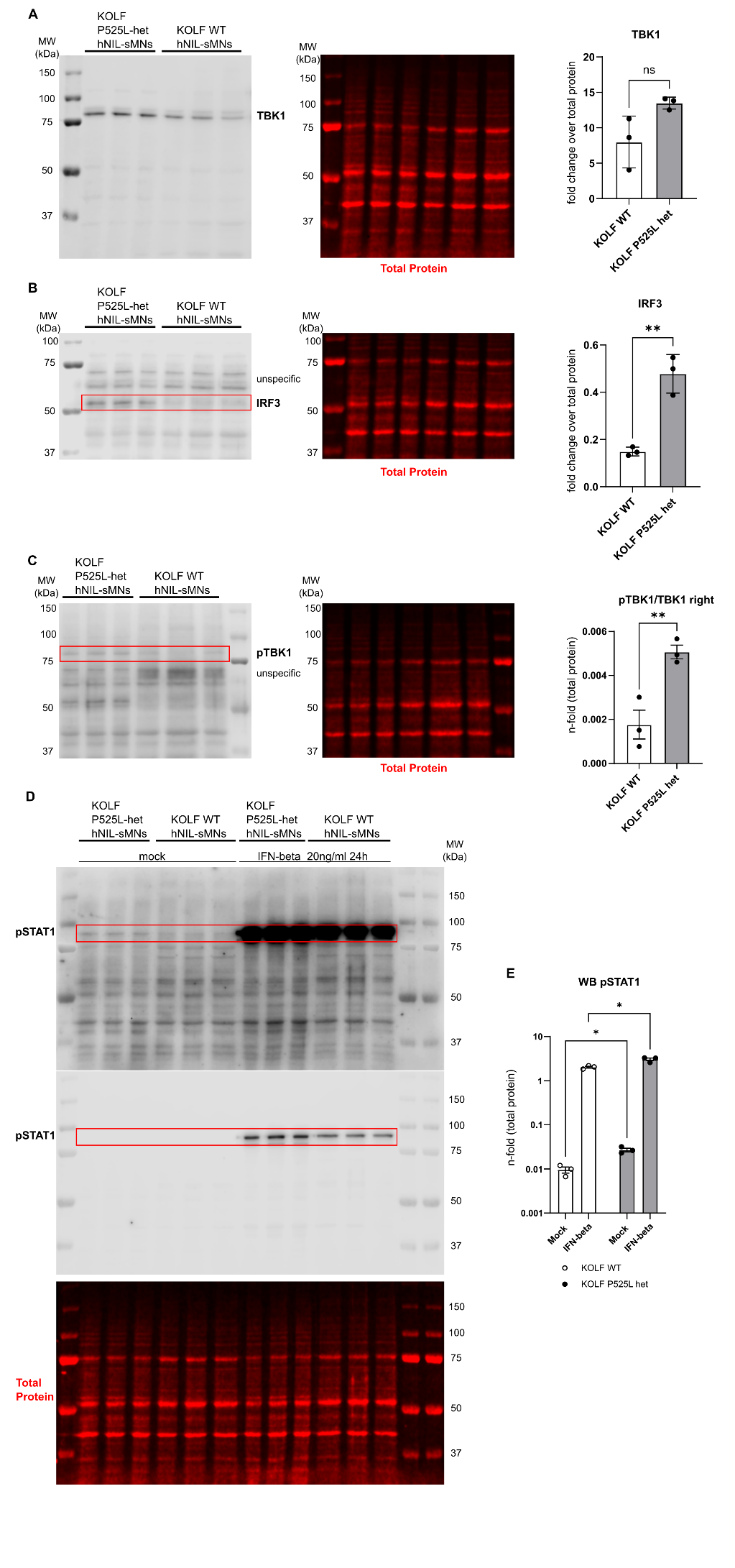
**

**Supplement Figure 3: WB for TBK1, pTBK1, IRF3 and pSTAT1 in KOLF P525L-het hNIL-sMNs at day 10 after differentiation induction compared to isogenic WT:** WB of (A) TBK1 (~80kDa), (B) IRF3 (~55kDA), and (C) pTBK1 (~80kDa) for protein samples of the KOLF hNIL-sMN, including the respective total protein staining and quantification. Note the general high signal of IRF3 in FUS mutant sMN, which is only very faint in the KOLF WT. Similarly, pTBK1 is hardly recognizable in WT KOLF hNIL-sMN (high staining background here and unspecific bands below 75kDa). Quantification of (B-C) indicated a significantly higher signal of IRF3 and pTBK1/TBK1 in KOLF FUS-mutant hNIL-sMN, n=3 biological replicates, unpaired t-test. In this experiment, there was also a moderately, but not significantly lower TBK1 signal in the KOLF WT hNIL-sMN (A), unpaired t-test, n=3). (D) WB membrane stained against pSTAT1 for samples of KOLF WT and FUS P525L-het hNIL-sMN including total protein staining. To verify the antibody specificity, IFN-beta treatment was performed (20ng/ml, 24h), which resulted in a very strong and robust signal at approximately 90kDa (lane 8-13). However, on the same size level, there was also a faint signal for pSTAT1 detectable in untreated FUS-P525L hNIL-sMN samples (lane 2-4), which was hardly evident in the WT (lane 5-7). To facilitate interpretation, the raw 16-bit image of the membrane is visualized in two forms. Both originate from the same unprocessed dataset; the difference lies solely in the intensity-mapping strategy used for display to show that there was no oversaturation of the raw image. The membrane is shown twice to illustrate the quantification of (E) indicating a significantly higher pSTAT1 signal in the mutant hNIL-sMN, which is also evident after treatment with IFN-beta, indicating an involvement of the JAK-STAT pathway. Unpaired t-test, n=3.

**
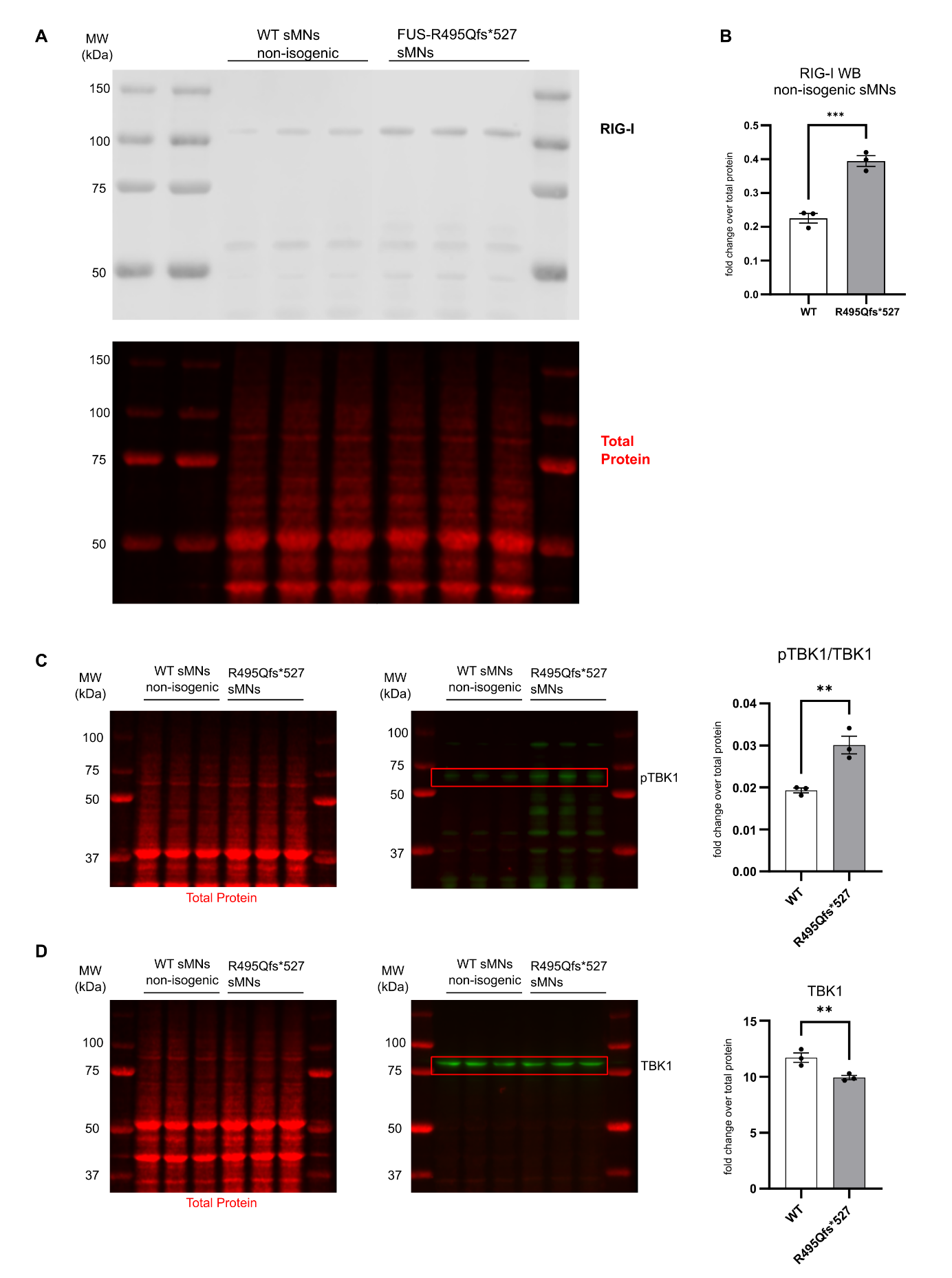
**

**Supplement Figure 4: WB staining for RIG-I in the non-isogenic WT/R495Qfs*527 sMN pair.** (A) WB membrane stained with an RIG-I antibody and corresponding total protein staining. At the expected bad size of approximately 102 kDA there was a stronger signal detectable in the R495Qfs*527 sMN compared to WT. (B) Quantification of (A) demonstrating a significantly higher abundance of RIG-signal in the R495Qfs*527 sMN compared to non-isogenic WT sMN, unpaired t-test, n=3 biological replicates. (C-D) TBK1 and pTBK1 WB in non-isogenic WT/R495Qfs*527. Significantly higher pTBK1 signal normalized to full TBK1 in the FUS^mut^ sMN. There was also a modest, but significantly lower TBK1 in FUS^mut^ sMN. N=3 biological replicates, unpaired t-tests.


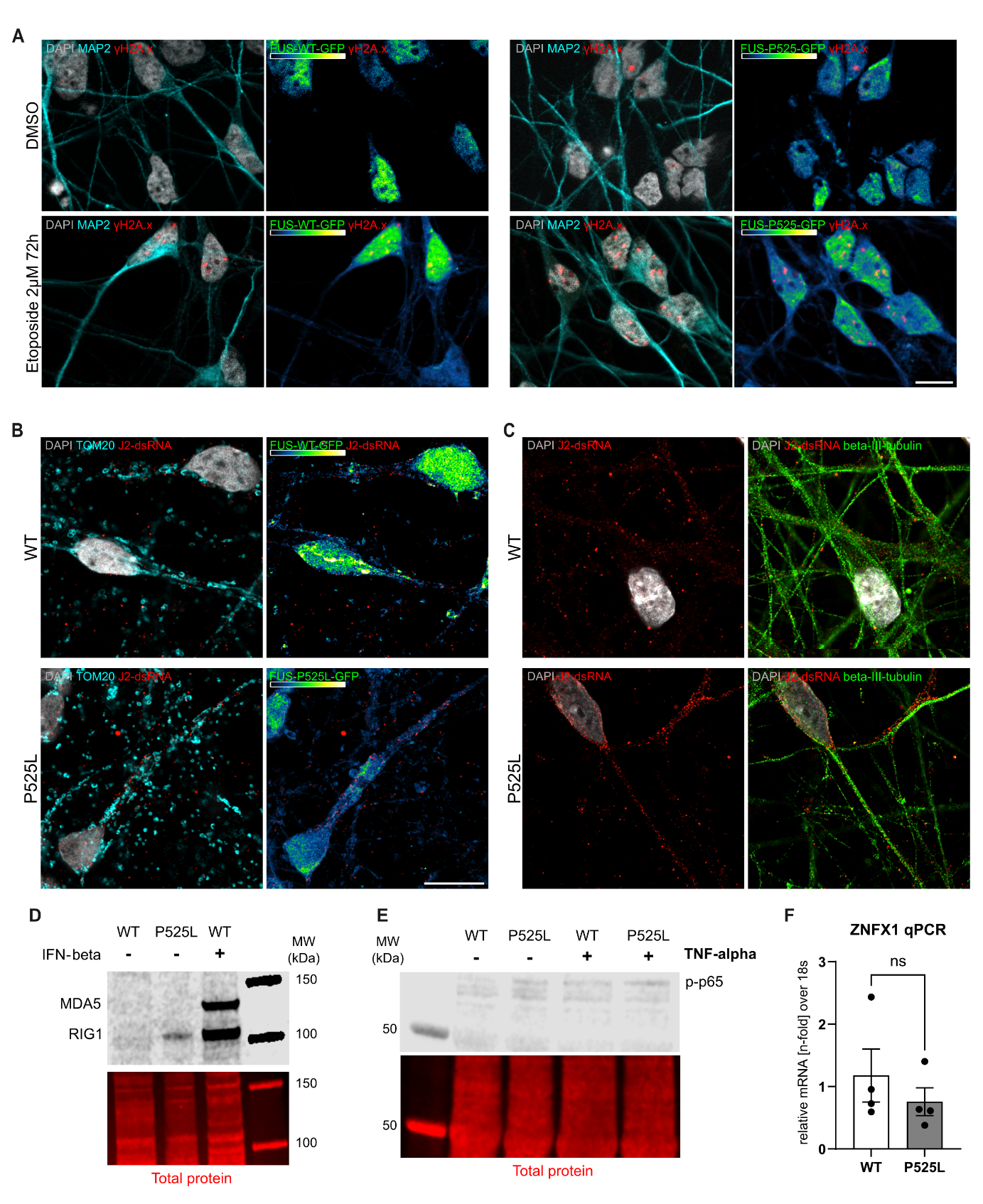


**Supplement Figure 5**: (A) IF panel of FUS^wt^ and FUS-P525L sMNs indicating yH2A.x as a measure of DNA double-strand breaks, scale bar 10µM. Etoposide 2µM 72h was used as a positive control. Note the increased amount of yH2A.x nuclear foci in FUS^mut^ sMNs at baseline. The green-blue-fire LUT for FUS-GFP was included to illustrate the cellular GFP low intensity background. (B) IF image panel of FUS^wt^ and FUS-P525L sMNs according to Fig. 2A. Cells were stained against the mitochondrial outer membrane protein TOM20 and the anti-dsRNA (J2) antibody. Nuclear staining was done with DAPI. C-terminally tagged FUS-GFP indicates FUS presence in the different conditions and is visualized via the Green-Fire-Blue LUT in Fiji. The green-blue-fire LUT for FUS-GFP was included to illustrate the low-intensity GFP background in cells. (C) IF panel indicating the usage of beta-III-tubulin as a neuronal marker, which was used as a mask for the majority of J2-dsRNA assessments. Cells were counterstained with DAPI. (D) WB scan for MDA5 and RIG-I in sMNs with either FUS^wt^ or FUS-P525L mutation. Treatment with IFN-beta 100ng/ml 24h as a positive control. At baseline, no signal for MDA5 was detected in either cell line, in contrast to RIG-I. (E) WB scan for phospho-p65 (p-RelA) in FUS^wt^ or FUS-P525L sMNs protein samples. TNF-alpha 20ng/ml for 24h was used as a positive control, indicated by the slight increase in band intensity on the third and fourth lanes. (F) qPCR of ZNFX1 mRNA, encoding another cellular sensor of dsRNA. Compared to the isogenic control sMN, FUS P525L sMN showed a non-significant trend towards reduced levels, unpaired t-test, n=4 biological replicates).

**
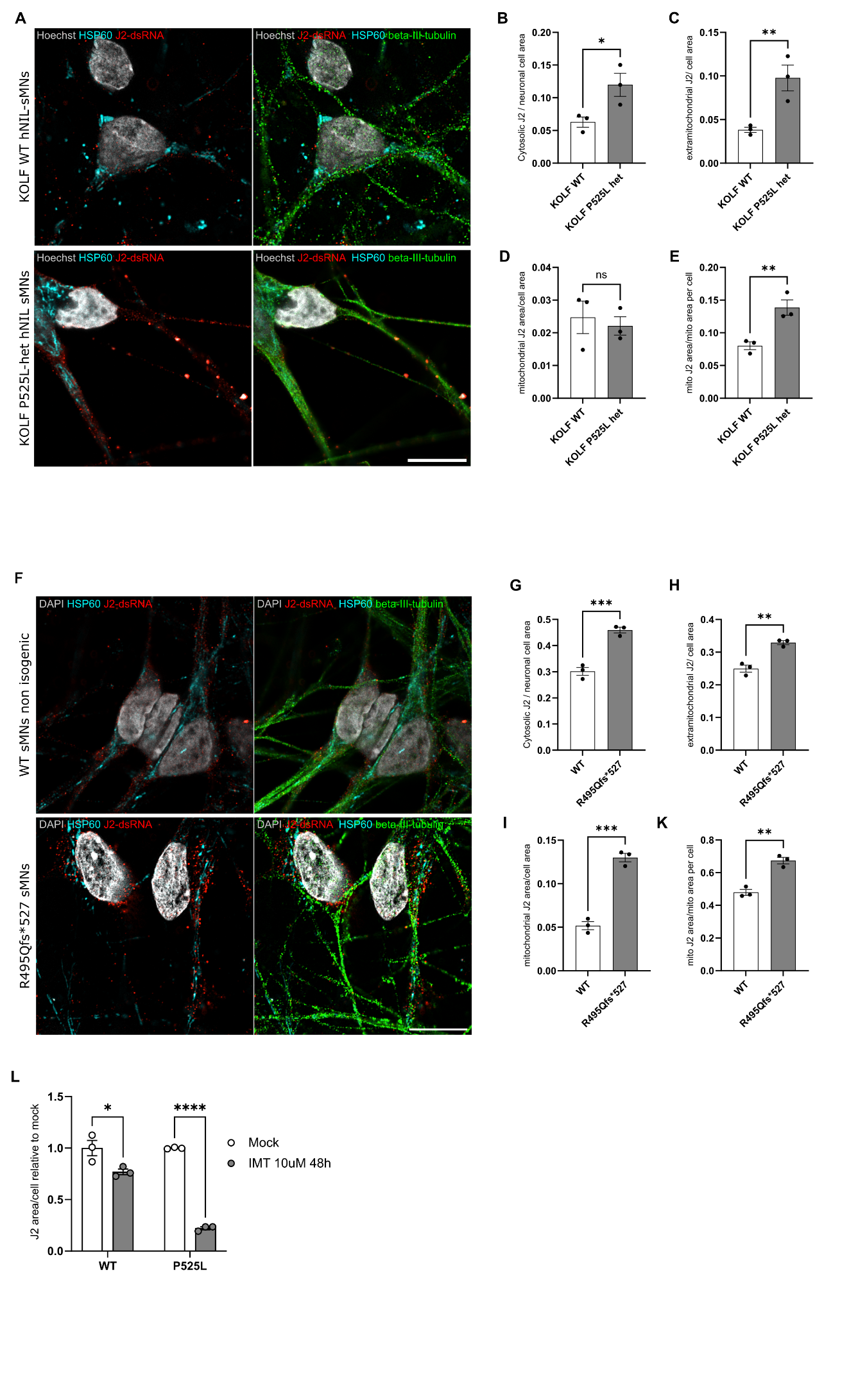
**

**Supplement Figure 6: J2-dsRNA IF analysis in KOLF WT/FUS P525L-het hNIL-sMN and in the non-isogenic WT/R495Qfs*527 sMN pair.** (A) IF panel of KOLF WT/FUS P525L-het hNIL-sMN demonstrating the IF of J2-dsRNA signal in neurons (beta-III-tubulin) and its partial overlay with mitochondria (HSP60) labelled in light blue. Scale bar = 10µM. (B-E) Quantification of J2-dsRNA in (A) and normalisation to single neurons (beta-III-tubulin mask) in B-D or to HSP60 (E). Different cellular compartments were defined by co-staining with HSP60 as a mitochondrial marker and removal of nuclei by subtraction of the Hoechst mask. As a result, J2-dsRNA was measured in the whole cytosol (B), only in the cytosol without mitochondria (C), and in mitochondria (D-E). In all compartments, there was a significantly higher staining signal found for J2-dsRNA in the KOLF FUS P525L hNIL-sMN compared to the isogenic WT. Unpaired t-test, n=3. (F) Similar to (A), IF panel of the non-isogenic WT/R495Qfs*527 sMN pair demonstrating the IF of J2-dsRNA signal in neurons (beta-III-tubulin) and its partial overlay with mitochondria (HSP60) labelled in light blue. Scale bar = 10µM. (G-K) quantification of the J2-dsRNA IF intensity in WT or R495Qfs*527 sMN as described for (B-E). In summary, there was also a significantly higher staining signal evident for J2-dsRNA in the R495Qfs*527 sMN compared to the non-isogenic WT sMN in all cytosolic compartments. Unpaired t-test, n=3 biological replicates. (L) Quantification of J2 IF signal in WT and FUS-P525L sMN per cell after IMT1 treatment (10µM 48), unpaired t-test, n=3. Normalization was done to the respective mock control condition (DMSO) and relative decrease is shown.

**
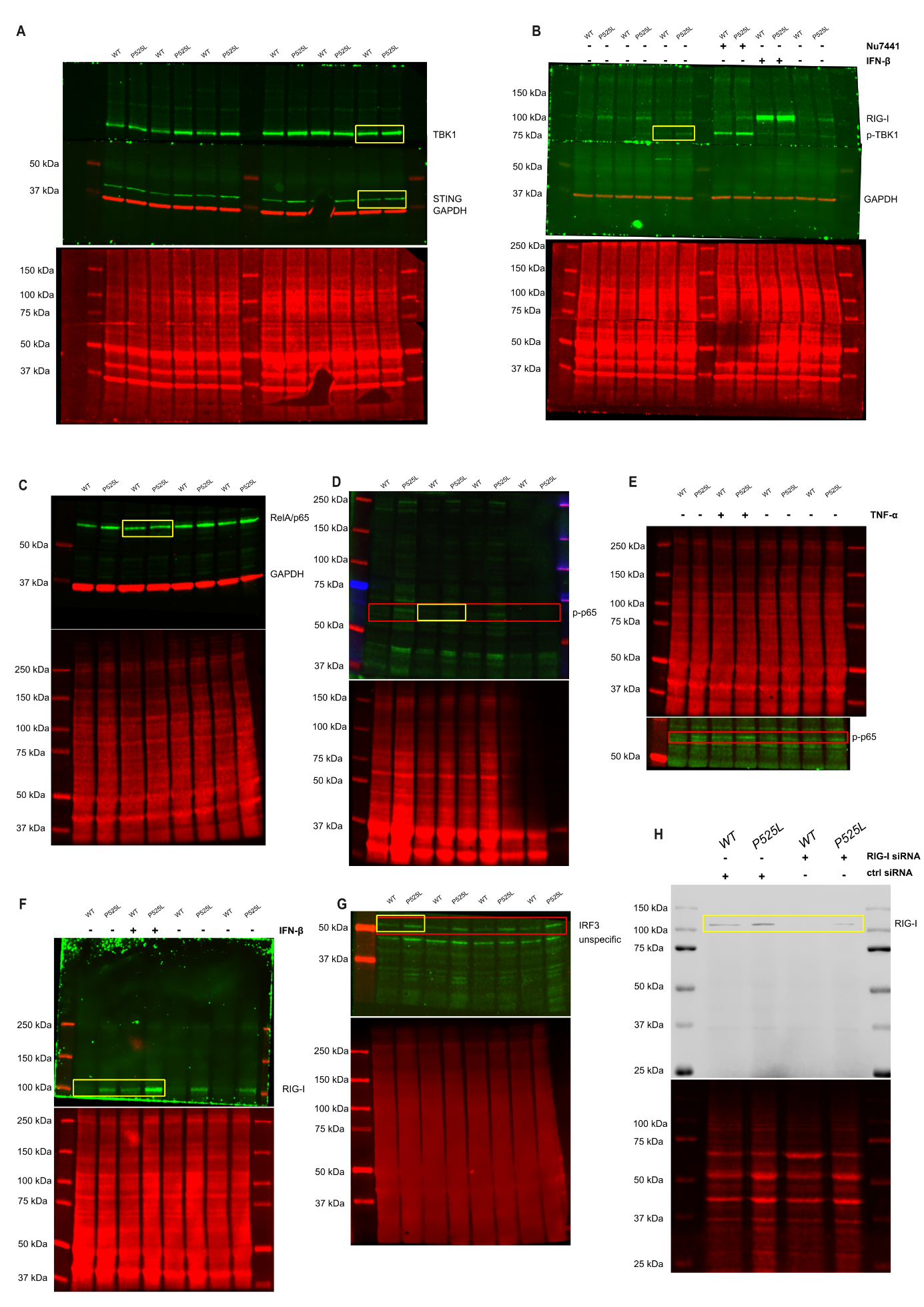
**

**Supplement Figure 7:** Raw WB images corresponding to cropped WB images in Figure 1, 3 and Suppl. Figure 1 (indicated by yellow rectangles). (A): Raw western blot membrane for Fig. 1D - TBK1 & STING insets. This signal on this membrane was normalized to the total protein staining provided below and used for quantification, depicted in 1E and 1H. (B) Raw western blot membrane for inset in Fig. 1D (pTBK1-signal) and RIG-I raw signal, which was used for the quantification in Fig. 3D. Note the positive control treatment conditions IFN-beta for RIG-I antibody detection and the DNA-PK inhibitor Nu4771 for pTBK1 signal. (C) Raw western blot membrane for Fig. 1D – p65/RelA inset, which was also used for the quantification in Fig. 1I. (D) Raw western blot membrane for Fig. 1D – pRelA inset, which was also used for the quantification in Fig. 1K. (E) Raw western blot membrane for Suppl. Fig. 1E demonstrating the use of TNF-alpha as a positive to induce a pRelA signal. (F) Raw western blot membrane for Fig. 3C stained with an RIG-I antibody and treatment with IFN-beta as a positive control for signal induction of RIG-I. (G) Raw western blot membrane for Fig. 1D – IRF3 inset, which was also used for the quantification of in Fig. 1G. (H) Raw western blot membrane for Fig. 3H stained with an RIG-I antibody showing the effect of RIG-I/DDX58 knockdown with siRNA (Lane 4+5).

**Supplemental Table 1:** Patient/proband characteristics

| **Genotype** | **Cell line** | **Sex** | **Age at biopsy** | **Mutation** | **Motoneuron characterization in** |
| --- | --- | --- | --- | --- | --- |
| Wt | Ctrl1 | female | 43 | - | [1] |
| Wt | Ctrl2 | female | 49 | - | [1] |
| IGC | FUS-WT eGFP^het^ | isogenic to FUS R521C and FUS-P525L GFP | N/A | - | [2] |
| Mt | FUS R521C^het^ | female | 58 | R521C | [3] |
| Mt | FUS-P525L eGFP^het^ | isogenic to FUS R521C and FUS-WT GFP | N/A | P525L | [2] |
| Mt | FUS R521L | Female | 65 | R521L | [3] |
| Mt | FUS R495Qfs*527 | male | 29 | R495QfsX527 | [3] |
| Wt | KOLF WT | - | - | - | This work |
| Mt | FUS-P525L heterozygous | - | - | P525L heterozygous | This work |
|  |  |  |  |  |  |
|  |  |  |  |  |  |

**Supplemental Table 2.** Post mortem examined in this study.

| **Case No.** | **Age** | **Gender** | **Cause of death** | **Clinical diagnosis** | **Pathological**  **diagnosis** | **pTDP-43** |
| --- | --- | --- | --- | --- | --- | --- |
| 1 | 61 | F | pneumonia | - | ***Normal*** | nd |
| 2 | 72 | F | myocardial infarction | - | ***Normal*** | nd |
| 3 | 70 | F | respiratory | R521C sALS | ***FUS*** | - |
| 4 | 40 | F | pneumonia | R521C fALS | ***FUS*** | - |

PMI = Postmortem interval. Nd = no data available

**References**

[1] P. Reinhardt, M. Glatza, K. Hemmer, Y. Tsytsyura, C. S. Thiel, S. Hoing, S. Moritz, J. A. Parga, L. Wagner, J. M. Bruder, G. Wu, B. Schmid, A. Ropke, J. Klingauf, J. C. Schwamborn, T. Gasser, H. R. Scholer, J. Sterneckert, *PLoS One* **2013**, *8* (3), e59252, <https://doi.org/10.1371/journal.pone.0059252>.

[2] M. Naumann, A. Pal, A. Goswami, X. Lojewski, J. Japtok, A. Vehlow, M. Naujock, R. Gunther, M. Jin, N. Stanslowsky, P. Reinhardt, J. Sterneckert, M. Frickenhaus, F. Pan-Montojo, E. Storkebaum, I. Poser, A. Freischmidt, J. H. Weishaupt, K. Holzmann, D. Troost, A. C. Ludolph, T. M. Boeckers, S. Liebau, S. Petri, N. Cordes, A. A. Hyman, F. Wegner, S. W. Grill, J. Weis, A. Storch, A. Hermann, *Nature communications* **2018**, *9* (1), 335, <https://doi.org/10.1038/s41467-017-02299-1>.

[3] J. Japtok, X. Lojewski, M. Naumann, M. Klingenstein, P. Reinhardt, J. Sterneckert, S. Putz, M. Demestre, T. M. Boeckers, A. C. Ludolph, S. Liebau, A. Storch, A. Hermann, *Neurobiology of disease* **2015**, *82*, 420, <https://doi.org/10.1016/j.nbd.2015.07.017>.
